# A sustainable and effective bioprocessing approach for improvement of acid phosphatase production and rock phosphate solubilization by *Bacillus haynesii* strain ACP1

**DOI:** 10.1038/s41598-022-11448-6

**Published:** 2022-05-27

**Authors:** Soad A. Abdelgalil, Mohamed M. Y. Kaddah, Mahmoud E. A. Duab, Gaber A. Abo-Zaid

**Affiliations:** 1 Bioprocess Development Department, Genetic Engineering, and Biotechnology Research Institute (GEBRI), City of Scientific Research and Technological Applications, New Borg El-Arab City, Alexandria, 21934 Egypt; 2 Pharmaceutical and Fermentation Industries Development Center, City of Scientific Research and Technological Applications, New Borg El-Arab City, Alexandria, 21934 Egypt

**Keywords:** Environmental biotechnology, Industrial microbiology, Applied microbiology, Environmental microbiology, Fungi, Biocatalysis, Biochemistry, Biotechnology, Microbiology, Microbiology techniques

## Abstract

There is indeed a tremendous increase in biotechnological production on a global scale, more and more innovative bioprocesses, therefore, require to perform ideally not only in a small lab- but also on large production scales. Efficient microbial process optimization is a significant challenge when accomplishing a variety of sustainable development and bioengineering application objectives. In Egypt's mines, several distinct types of rock phosphate (RP) are utilized as a source of phosphate fertilizers in agriculture. It is more ecologically beneficial to utilize RP bio-solubilization than acidulation. Therefore, this work aimed to strategically scale up the acid phosphatase (ACP) production and RP bio-solubilization by the newly-discovered *Bacillus haynesii*. The use of consecutive statistical experimental approaches of Plackett–Burman Design (PBD), and Rotatable Central Composite Design (RCCD), followed by pH-uncontrolled cultivation conditions in a 7 L bench-top bioreactor revealed an innovative medium formulation. These approaches substantially improved ACP production, reaching 207.6 U L^−1^ with an ACP yield coefficient *Y*_*p/x*_ of 25.2 and a specific growth rate (*µ*) of 0.07 h^−1^. The metals Na, Li, and Mn were the most efficiently released from RP during the solubilization process by *B. haynesii.* The uncontrolled pH culture condition is the most suitable setting for simultaneously improving the ACP and organic acids production. The most abundant organic acid produced through the cultivation process was lactic acid, followed by glutamic acid and hydroxybenzoic acid isomer. The findings of TGA, DSC, SEM, EDS, FTIR, and XRD analysis emphasize the significant influence of organic acids and ACP activity on the solubilization of RP particles.

## Introduction

Today's globalization has forced sustainable agriculture to fulfill our agricultural requirements, which conventional agriculture cannot. Agricultural production systems must be intensified to support productivity gains and revenue generation to maintain food security in developing countries. Among the necessary macronutrients for higher plants and animals is phosphorus (P), whereas, numerous metabolic pathways require the presence of P to operate. Egypt has alkali soils that make phosphate minerals exceedingly challenging to dissolve. Using RP as a source of P in the right way can contribute to sustainable agricultural intensification, especially in developing countries with RP deposits^1^. In mineralogy and geology, phosphates are rocks or ore that contain phosphate ions. Phosphorite, also known as RP, is an exhaustible nonrenewable natural resource of a non-detrital sedimentary rock that includes substantial quantities of phosphate-bearing minerals^2^. Different geological rocks may be found across the world.

RP, as well as upgraded phosphates derived from it, are significant minerals in the world. Ninety-seven percent of the world's phosphate ore production is concentrated in 16 countries, each producing millions of tons each year. The phosphate can be provided in the form of hydroxyapatite [Ca_10_(PO_4_)_6_(OH)_2_], fluorapatite [Ca_10_(PO_4_)_6_F_2_], carbonate apatite [3Ca_3_(PO_4_)_2_·CaCO_3_], and sulpho-apatite [3Ca_3_ (PO_4_)_2_·CaSO_4_]. Igneous and metamorphic apatites are typically thought to be less reactive because of their well-developed crystalline structure. Egyptian phosphorite deposits are discovered as beda, layers, and lenses intercalated with limestones, chert, claystone, and marl bed deposits^3^. Currently, only a few RP deposits are mined; about 90 percent of the world's P fertilizer output comes from RP mining. The rest is used in animal feed, detergents, and chemicals. Although the soil may be treated with RP directly on the surface, its access to plants is sluggish^2^. In recent years, a great deal of study has been done on creating innovative, ecologically, and friendly approaches for RP solubilization, as opposed to the traditional mineral acid procedure. Commercial bio-inoculants and large-scale bioprocessing of RP utilizing phosphate-solubilizing microorganisms have resulted in highly efficient, low-cost, and successful commercial alternatives that are currently utilized by the agro-industry all over the world^4^.

Mineralization processes to break down RP are accomplished by various microbiological activities, including organic acid production, proton extrusion, and phosphatase enzyme^5^. Phosphatases are a family of enzymes that hydrolyze phosphoesters (R-O-PO_3_) in various substrates under varied circumstances. Phosphatase may be divided into various groups based on characteristics like specificity and optimal pH; one of these families is the bacterial non-specific acid phosphohydrolases or acid phosphatases^6^. Bacterial acid phosphatases (EC 3.1.3.2) are diverse enzymes that may dephosphorylate a wide variety of structurally unrelated organic phosphoesters to get inorganic phosphate (P_i_) and organic byproducts and exhibit optimal catalytic activity at acidic to neutral pH values. They are released as soluble periplasmic proteins or retained as membrane-bound lipoproteins. When specific phosphate acceptors are present, these enzymes catalyze the hydrolysis of a variety of phosphomonoesters and transphosphorylation reactions by transferring a phosphoryl group to alcohol^7^. Although the specific function of bacterial ACP is not well known, it is widely recognized as a scavenger of organic phosphate esters that would otherwise be unable to get through the cytoplasmic membrane. ACPs have also been used in agriculture to promote plant growth and bioremediation, metal recovery, and even as an enzyme reporter in enzyme immunoassays, according to certain publications^8–10^.

ACPs are found in both eukaryotic and prokaryotic species, and great attention has been paid to these enzymes to understand their structures, functions, and catalytic mechanisms. However, there are so few bacteria in the biosphere that can solubilize phosphate. There is tremendous competition for phosphate among microorganisms, even though many species produce phosphatases and have elaborate mechanisms for regulating their production and activity^11^. Therefore, industries are still seeking novel bacterial species capable of producing industrial enzymes to meet the current ACP enzyme demand. Appropriate selection of various industrial microorganisms and optimizing fermentation conditions are required to generate low-cost industrial enzymes. The production of microbial industrial enzymes under optimal conditions to get enzymes with acceptable characteristics is a never-ending process.

A fermentation medium's composition can considerably impact product concentration, yield, and volumetric productivity when establishing an industrial fermentation. The decision of how to invest limited experimental resources is a critical aspect of scientific investigation^12^. Therefore, the invention of predictive mathematical models opens up a whole new world of possibilities for the logical design of microbial product manufacturing. The design of experiments (DoE) approach formalizes the use of mathematical models to determine the most informative trials and gives strategies for avoiding numerous traps that trial-and-error experimentation might fall into. Moreover, much attention has been paid to developing clean and sustainable alternatives to fossil-based materials through bioprocessing. Scaling up fermentation processes is crucial in bringing new technologies and bioproducts to market in the biotech sector. Kinetic models that predict cell behavior in dynamic external settings and fluid dynamics models that explain mass transfer and mixing in the bioreactor may be used to predict the output of the whole culture system at different scales for reasonable bioprocess scale-up^13^.

**Figure 1:**
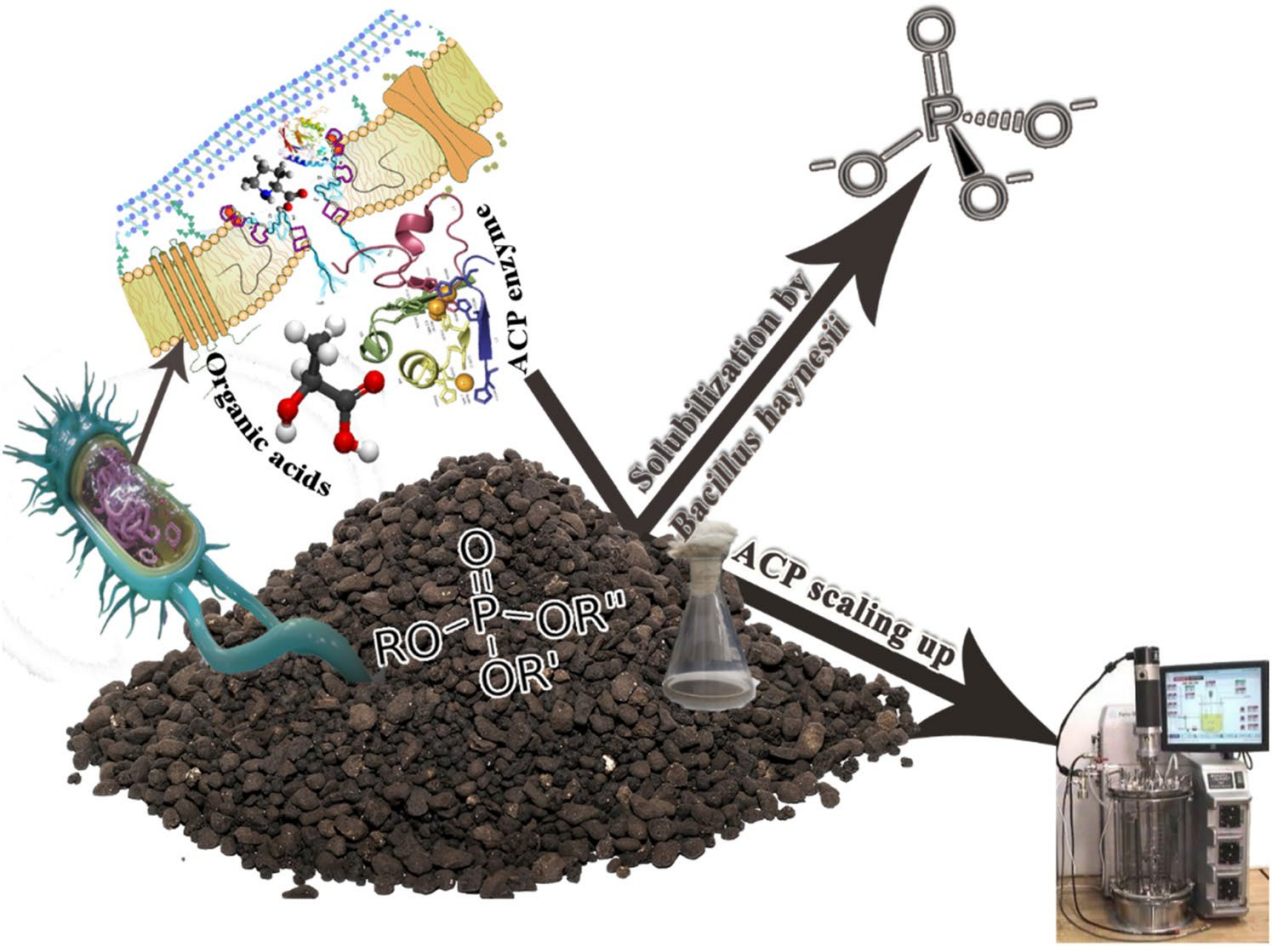
Schematic diagram showing the ultimate benefit utilization of characteristic features of new-discovered *B. haynesii* strain ACP1 for bio-solubilization of RP and boosting the production of ACP on bench-top bioreactorscale.

A few reports on RP solubilization by various bacteria are available^5^. A study on the use of natural minerals like RP to scale up the production of bacterial ACP for solubilization has not been published yet. In addition, a new challenge is the simultaneous production of industrial ACP enzymes from microorganisms in a single economic production medium. The present contribution aims to shed a light on the performance evaluation of a novel conceptual bioprocess for simultaneous ACP production with RP bio-solubilization utilizing recently discovered *Bacillus haynesii* strain ACP1, as illustrated in Fig. 1. Based on the authors' knowledge, It also represents a significant step forward in scaling up ACP production from shake-flask to bench-top bioreactor scale.

## Results and discussion

### Isolation and identification of ACP-producing bacteria

From the isolation strategies program, the forty-five isolates representing different morphotypes were isolated from the collected wastewater samples of the Alexandria tanning and leather factories. The isolate entitled ACP1 manifested the highest obviousness colonies with a clear visible zone on Pikovskaya and CaCO_3_ agar plates. It showed a bright yellow colony growth on the *p*NPP-rich medium with the most significant ACP activity of 54 U L^−1^ min^−1^ among the selected isolates. ACP1 was thus chosen for a more in-depth study. The present finding is corroborated by Sakurai et al.^14^, who discovered a significant correlation between phosphatase activity and phosphate solubilizing capabilities. The obtained *16S rRNA* sequence (1392 bp nucleotide sequence) of ACP1 was submitted to a BLAST search of the GenBank database. BlastN was used to match the resultant *16S rRNA* gene sequence to other *16S rRNA* gene sequences available in GenBank (Augest 2021). The study revealed that strain ACP1 was phylogenetically related to members of the genus *Bacillus* with sequence identities to *B. haynesii* of 99.64 with a query cover of 100%. Also, the isolate shares sequence similarities of either 99.43 or 99.35% with the remaining *Bacillus* type strains like *B. licheniformis*, and *B. sonorensis*; respectively.

**Figure 2:**
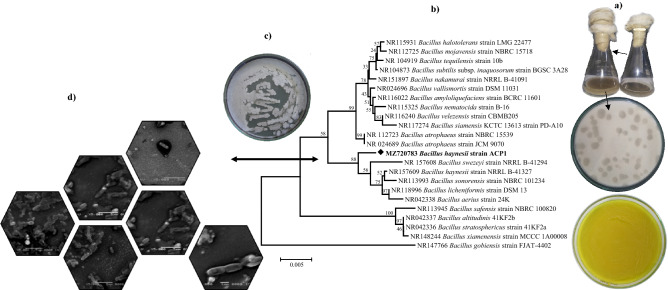
Isolation, screening, and identification of phosphate solubilizing bacteria (PSB) and acid phosphatase-producing bacteria, **(a)** isolation and qualitative screening of PSB and ACP-producing bacteria, **(b)** phylogenetic tree based on *16S rRNA* gene sequence analysis showing the relationship of *B. haynesii* strain ACP1 with reference strains (NCBI GenBank) constructed using the neighbor-joining method with the aid of MEGA 7.0 program, **(c)** cultural feature of *B. haynesii* strain ACP1 agar plate, and **(d)** SEM micrograph of *B. haynesii* strain ACP1 showing cell morphology at a magnification of ×5000 and 10,000 with 15 kV.

For comparative analysis between strain ACP1 and its closest relatives, a phylogenetic tree was constructed by Mega X software package using a neighbor-joining algorithm. The resultant tree topologies (Fig. 2b) show that the isolate forms a well-supported branch in the *Bacillus 16S rRNA* gene tree. It was noticed that the genus *Bacillus* forms three significant clusters, while strain ACP1 forms a separate clade together with other isolates. The type strain of *B. licheniformis* joins this group, albeit with low bootstrap support (88%). So, the position of strain ACP1 within the evolutionary tree demonstrates a unique lineage. This phyletic line, together with *B. haynesii* strain NRRL B-4132, was consistently found in the same clade. Because the strain ACP1 closely resembled *B. haynesii* based on taxonomic characteristics, it was given the proposed name *B. haynesii* strain ACP1. Microscopically characterization of the strain ACP1 revealed gram-positive bacteria with rod-shaped cells of about 1.5–2.10 µm long and 0.7–0.87 µm wide and spore-forming bacteria. Moreover, morphologically it is characterized by an irregular colony shape on agar media with irregular (undulate, fimbriate) margins. The colonies' surfaces are often rough and wrinkled with hair-like growth, as shown in (Fig. 2c,d).

Several studies have looked at the capacity of various bacteria to dissolve insoluble organic phosphate complexes such as tricalcium phosphate (TCP), dicalcium phosphate (DCP), hydroxyapatite, and RP^15^. *Pseudomonas*, *Leclercia*, *Piriformospora, Serratia*, *Burkholderia*, *Pantoea*, *Citrobacter*, *Rhizobium*, and *Enterobacter* are only a few of the bacterial species possessing phosphate solubilization along with ACP production capabilities^5,8,16,17^. To the authors’ knowledge, there are no previously published reports on the scaling up of ACP production accompanied by phosphate solubilizing features from *B. haynesii.*

### Traditional optimization of the physical parameters for ACP production

It was noticed from the present study that the *B. haynesii* strain ACP1 yielded maximum ACP production (40.8 U L^−1^) at 50 °C. A lower than optimal temperature (45 °C) impedes chemicals' transit across cells, reducing ACP production (34.6 U L^−1^). Due to denaturation and conformational changes of the enzyme, ACP production gradually decreased reaching 27.10 U L^−1^ at temperatures above the optimal 55 °C. Although, 45 °C was recorded as the choicest temperature for the maximal ACP production (77.87 U mL^−1^) by *Serratia* sp., and beyond the optimal temperature, the ACP production trend decreased, contradictory to the present finding^8^. In the current study, it was observed that there is a gradually incrementing in the ACP production trend from 37.2 U L^−1^ (at pH 3.0) to reaching its optimal activity peak of 49 U L^−1^ at pH 7.0 passing by 39.4 U L^−1^ (at pH 4.0), 40.7 U L^−1^ (at pH 5.0), and 44.4 U L^−1^ (at pH 6.0). The declining ACP throughput (47.7 U L^−1^) was noticed beyond the optimal pH value at pH 8.0. This finding was congruent with earlier observations of Musarrat et al.^8^, who recorded that the neutral or slightly acidic pH was shown to be optimal for bacteria to solubilize organic phosphate. On the other hand, the results elucidated that the initial pH unadjusted media (approximately pH 7.5) shows the best and the optimal condition for maximal ACP production (56.9 U L^−1^) rather than the adjusted one with 1 N HCl (49 U L^−1^). As follows, unadjusted pH media was used throughout all further studies under investigation in the current study. Although Behera et al.^18^ noted that the maximum production of ACP from *Serratia* sp. was achieved at pH 5 (80.66 U mL^−1^) and decreased after that, this finding is paradoxical with the conclusions obtained. Additionally, the optimal inoculum size of the activated pre-culture obtained in this investigation for the maximal production of ACP (64.1 U L^−1^) is 4.0% (v/v), there was a drop-down in ACP production as the inoculum size increased, ACP throughput was reached 56 U L^−1^ when 10% of inoculum size used.

### Statistical optimization of ACP production by *B. haynesii* strain ACP1

To prescreen the influence of eleven different medium constituents namely glucose, potassium citrate, NaNO_3_, (NH_4_)_2_SO_4_, urea, RP, NaCl, MgCl_2_•6H_2_O, CoCl_2_•6H_2_O, CuSO_4_•5H_2_O, NiSO_4_, corresponding to *X*_*1*_–*X*_*11*_, respectively, at their lowest and highest factor levels on ACP production, a structured experimental design matrix with sixteen trials was developed using the PBD. As notable from Table 1, ACP exhibited broad variation in terms of its activity throughout the design matrix, highlighting the importance of medium optimization in boosting ACP efficiency. Both RP (*X*_*6*_) and glucose (*X*_*1*_) are essential for the promotion of the synthesis of ACP since the most significant ACP activity (66.5 U L^−1^) was found in trial number 1, which included high concentrations of both nutritional media (20, and 10 g L^−1^, respectively) as seen in Table 1. The drop-down of ACP productivity (35.9 U L^−1^) was observed when the minor concentration of both media constitutes (2, and 1 g L^−1^, respectively) was used, which that achieved in experiment number 10, and this, in turn, reflects the significance of RP and glucose in enhancement the ACP throughput.

**Table 1:** Randomized Plackett–Burman experimental design for evaluating factors influencing ACP production by *B. haynesii* strain ACP1.

Trails	Variables											ACP productivity (U L^−1^ min^−1^)		
	*X*_1_	*X*_2_	*X*_3_	*X*_4_	*X*_5_	*X*_6_	*X*_7_	*X*_8_	*X*_9_	*X*_10_	*X*_11_	Actual value	Predicted value	Residual
1	1	− 1	− 1	1	− 1	1	1	1	− 1	− 1	− 1	66.5239	66.0090	0.51498
2	1	1	1	1	1	1	1	1	1	1	1	40.4960	39.9810	0.51498
3	1	1	− 1	− 1	− 1	1	− 1	− 1	1	− 1	1	44.1312	43.6162	0.51498
4	1	− 1	1	1	1	− 1	− 1	− 1	1	− 1	− 1	45.5852	45.0703	0.51498
5	0	0	0	0	0	0	0	0	0	0	0	40.8595	42.4590	− 1.59948
6	0	0	0	0	0	0	0	0	0	0	0	41.0049	42.4590	− 1.45407
7	− 1	− 1	1	− 1	1	1	1	− 1	− 1	− 1	1	41.5138	40.9988	0.51498
8	0	0	0	0	0	0	0	0	0	0	0	40.8595	42.4590	− 1.59948
9	1	1	1	− 1	− 1	− 1	1	− 1	− 1	1	− 1	45.4398	44.9249	0.51498
10	− 1	1	− 1	− 1	1	− 1	1	1	1	− 1	− 1	35.9156	35.4007	0.51498
11	0	0	0	− 1	0	0	0	0	0	0	0	40.9322	42.4590	− 1.52678
12	− 1	− 1	1	− 1	− 1	1	− 1	1	1	1	− 1	36.6427	36.1277	0.51498
13	− 1	1	1	− 1	− 1	− 1	− 1	1	− 1	− 1	1	37.9513	37.4364	0.51498
14	− 1	− 1	− 1	− 1	− 1	− 1	1	− 1	1	1	1	38.4603	37.9453	0.51498
15	1	− 1	− 1	− 1	1	− 1	− 1	1	− 1	1	1	44.0585	43.5435	0.51498
16	− 1	1	− 1	− 1	1	1	− 1	− 1	− 1	1	− 1	38.9692	38.4542	0.51498

Variable			Code		Coded and actual levels									
					− 1		0		1 2					
Glucose			*X*_1_		1		5.5		10 10					
Pot. citrate			*X*_2_		0.1		0.55		1 0.5					
NaNO_3_			*X*_3_		0.1		0.3		0.5					
(NH_4_)_2_SO_4_			*X*_4_		0.1		0.3		0.5					
Urea			*X*_5_		0.1		0.3		0.5					
Rock phosphate			*X*_6_		2		11		20					
NaCl			*X*_7_		0.1		0.3		0.5					
MgCl_2_·6H_2_O			*X*_8_		0.02		0.06		0.1					
CoCl_2_·6H_2_O			*X*_9_		0.0005		0.0015		0.0025					
CuSO_4_·5H_2_O			*X*_10_		0.0005		0.0015		0.0025					
NiSO_4_			*X*_11_		0.0005		0.0015		0.0025					

### Mathematical multiple regression analysis of PBD results

**Figure 3:**
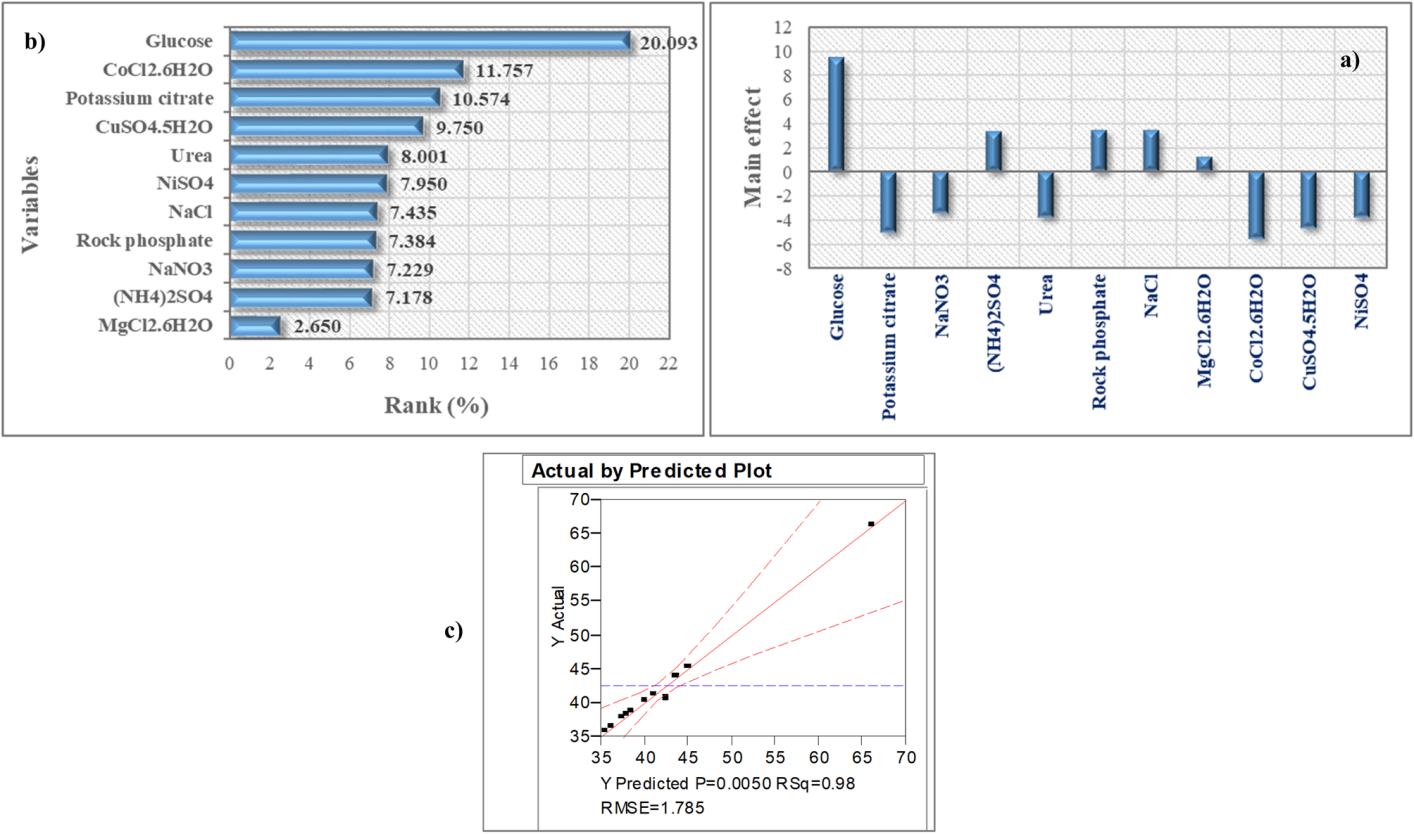
Plackett–Burman design results, **(a)** main effect chart of culture variables, **(b)** Pareto chart illustrating the order and significance of the variables affecting ACP production by *B. haynesii* strain ACP1 in a ranking percentage from 2.650 to 20.093, and **(c)** normal probability plot of the residuals for acid phosphatase production determined by the first-order polynomial equation.

The coefficient of multiple determinations (*R*^2^) and the lack-of-fit value can be used to determine the model's appropriateness, the resulting coefficients, *t*- and *p*-values are presented in Table 2. Table 2 and Fig. 3a illustrate the main effects of all independent factors on ACP production.

**Table 2:** Statistical analysis of Plackett–Burman design showing coefficient values, *t*- and *p*-values for each variable affecting ACP production.

Variables	Coefficient	Main effect	Std Error	*t*-Stat	*P*-value	Contribution %	Confidence level (%)
Intercept	42.45903		0.446244	95.14735	7.31551E^−08^		100
*X*_*1*_	4.731806	9.463614	0.515279	9.182993	0.0007809	20.0926	99.92190
*X*_*2*_	− 2.490105	− 4.98021	0.515279	− 4.83253	0.0084457	10.5737	99.15542
*X*_*3*_	− 1.702481	− 3.40496	0.515279	− 3.30399	0.0298215	7.22922	97.01784
*X*_*4*_	1.690363	3.38072	0.515279	3.28048	0.0304869	7.1777	96.95130
*X*_*5*_	− 1.884240	− 3.76848	0.515279	− 3.65673	0.0216424	8.0010	97.83575
*X*_*6*_	1.738833	3.477666	0.515279	3.37454	0.0279260	7.3835	97.20739
*X*_*7*_	1.750950	3.501900	0.515279	3.39806	0.0273261	7.43503	97.26738
*X*_*8*_	0.6240411	1.248082	0.515279	1.21107	0.2925245	2.64985	70.74754
*X*_*9*_	− 2.768803	− 5.537607	0.515279	− 5.37340	0.0057941	11.7571	99.42058
*X*_*10*_	− 2.296228	− 4.592457	0.515279	− 4.45628	0.0111923	9.75045	98.88076
*X*_*11*_	− 1.872123	− 3.744246	0.515279	− 3.63322	0.0220967	7.94957	97.79032

ANOVA	df	SS	MS	*F*	Significance *F*		
Regression	11	729.832	66.3483	20.82397	0.005		
Residual	4	12.7446	3.18615				
Total	15	742.576					
*R*^*2*^	0.982						
Adj. *R*^*2*^	0.935

Among different medium components investigated, glucose, (NH_4_)_2_SO_4_, RP, and NaCl exhibited a positive impact on the production of ACP, whereas, potassium citrate, NaNO_3_, urea, CoCl_2_•6H_2_O, CuSO_4_•5H_2_O, NiSO_4_ had quite a negative impact and MgCl_2_•6H_2_O had a minor impact on ACP productivity. As clear from Table 2, the confidence level, low *p*-value, and *t*-value of glucose (*X*_*1*_) are 99.9%, 0.00078, 9.182, respectively, with a contribution percent of 20.09, this signifies the significant impact of glucose on the production process. Ammonium sulfate (NH_4_)_2_SO_4_ was found significant at 96.9%, 0.03, and 3.28, respectively, with a contribution percent of 7.17. RP and NaCl had a confidence level of 97.2, 97.26% with a *p*-value of 0.0279, 0.0273, *t*-value of 3.37, 3.39, and contribution percent of 7.38, 7.43, respectively, they also were exerted their significant impact on the ACP production. Other coefficient terms employed in this model had no discernible impact on ACP output. This suggests that the ACP production may be substantially boosted by increasing the concentrations of glucose, (NH_4_)_2_SO_4_, RP, and NaCl in growth media, while ignoring the other insignificant factors.

Pareto chart Fig. 3b revealed that glucose (*X*_*1*_) was the most significant factor affecting ACP production with contributions (20.09%) followed by CoCl_2_•6H_2_O (*X*_*9*_), potassium citrate (*X*_*2*_), CuSO_4_•5H_2_O (*X*_*10*_), urea (*X*_*5*_), NiSO_4_ (*X*_*11*_), NaCl (*X*_*7*_), RP (*X*_*6*_), NaNO_3_ (*X*_*3*_), (NH_4_)_2_SO_4_ (*X*_*4*_), and then MgCl_2_•6H_2_O (*X*_*8*_).

The normal probability plot is a fundamental graphical approach for evaluating the model's accuracy. Using the current model, the normal probability chart as illustrated in Fig. 3c indicates that many residuals are near the fitted model's line. This shows that the model was able to predict the experimental findings accurately.

A coefficient of determination (*R*^*2*^) of 98.2% was observed using the current model. Only 1.8% of the variance could not be explained by the model, implying that 98.2% of the experimental data were consistent with the model. Furthermore, the high adjusted coefficient of determination value (Adj. *R*^*2*^ = 0.935) suggests the increased importance of the model and an appropriate correlation between the examined variables and ACP production. Moreover, the model *p-*value of 0.005 and *F*-value of 20.82 show the model's relevance, just only a 0.5% chance that a model *F*-value this large could occur due to noise.

#### Regression equation

ANOVA revealed that the first-order model explaining the correlation between the eleven factors studied across 16 trials and the ACP activity might be expressed as the equation:

$$\begin{aligned} Y_{activity} & = 42.45 + 4.73X_{1} - 2.49X_{2} - 1.70X_{3} + 1.69X_{4} \\ & \quad - 1.88X_{5} + 1.73X_{6} + 1.75X_{7} + 0.62X_{8} \\ & \quad - 2.76X_{9} - 2.29X_{10} - 1.87X_{11} \\ \end{aligned}$$

The optimal production medium conditions were predicted to be: (g L^−1^) glucose, 10; (NH_4_)_2_SO_4_, 0.5; RP, 20; and NaCl, 0.5 without pH adjustment, the cultivation conditions carried out at 50 °C, 200 rpm using 4.0% v/v activated inoculum size for 24 h.

Throughout these settings, the highest ACP activity was 65.4 U L^−1^, which is slightly higher than the activity achieved before using PBD (64.1 U L^−1^). But, through PBD strategies, a novel and unique formulation of nutritive media with low-cost and straightforward constituents was created for solubilizing organic P concordant with production ACP, even though the boosting of ACP production is not different from that obtained from the original media before optimization.

Behera et al^8^ demonstrated that, among the various carbon and nitrogen sources utilized in their investigation, glucose and (NH_4_)_2_SO_4_ were the best carbon and inorganic nitrogen sources for *Serratia* sp. growth and ACP production (80.66 and 80.92 U mL^−1^, respectively) which concurrence with the finding of the present study. To the authors’ knowledge, there are no reports on the production and optimization of bacterial ACP production using statistical and mathematical designs until now (Sept 2021). To the best of our knowledge, the current report is the first to deal with medium formulation by complicated statistical optimization strategies for boosting bacterial ACP productiveness on a laboratory scale to minimize the cost of the production process and enhance the organic phosphate solubilization process.

### Response surface methodology (RSM)

The empirical half-factorial RCCD was employed to build the RSM model. Based on the earlier PBD screening, the four studied variables (glucose, (NH_4_)_2_SO_4_, RP, and NaCl with confidence levels of 99.9, 96.9, 97.2, and 97.26%, respectively) were subjected to further investigation addressing their interaction and the modeling process. A precision random array of thirty experimental trials were conducted using the RCCD with eight axial, sixteen factorial, and six central points to optimize the chosen variables. The Codes and actual values of the picked variables, the design matrix, and experimental and predicted responses are illustrated in Table 3. Depending on the four independent factors, the efficacy of ACP activity varied significantly. For example, Table 3 shows that the highest ACP yield of ~ 115.16 U L^−1^ (predicted to be 114.7 U L^−1^) was obtained at zero level concentrations of the chosen variables in center point experimental trials (numbers 3 and 16). In contrast, the lowest ACP activity of 57.2 U L^−1^ (predicted to be 70 U L^−1^) was observed in trial 22 when all predictors were held at their center points (zero levels) except RP, which was provided at its lowest or axial level (− 2) with a concentration of 12.5 g L^−1^. This discovery highlights the critical role of RP in the induction of ACP synthesis from *B. haynesii* strain ACP1.

**Table 3:** Matrix designed for *B. haynesii* strain ACP1 rotatable central composite design.

StdOrder	Trials	Type	Variables				ACP productivity (U L^−1^ min^−1^)		
			*X*_1_	*X*_2_	*X*_3_	*X*_4_	Actual value	Predicted value	Residual
10	1	Axial	2	0	0	0	72.7038	72.5584	0.1454
29	2	Axial	0	− 2	0	0	69.5048	79.0532	− 9.5484
18	3	Center	0	0	0	0	115.162	114.726	0.4362
19	4	Factorial	− 1	− 1	1	− 1	87.8262	83.9123	3.9138
23	5	Factorial	− 1	− 1	− 1	− 1	80.5558	74.6789	5.8768
3	6	Center	0	0	0	0	114.290	114.726	− 0.4362
15	7	Center	0	0	0	0	114.87	114.726	0.1454
28	8	Factorial	1	1	− 1	− 1	67.7599	65.7121	2.0478
1	9	Factorial	1	− 1	− 1	− 1	84.3364	78.2898	6.0465
4	10	Factorial	1	1	1	− 1	87.2445	89.1954	− 1.9508
7	11	Factorial	− 1	− 1	− 1	1	90.1527	83.2337	6.9189
24	12	Center	0	0	0	0	114.581	114.726	− 0.1454
27	13	Center	0	0	0	0	114.290	114.726	− 0.4362
20	14	Factorial	1	1	− 1	1	82.5915	81.5373	1.0542
14	15	Factorial	− 1	1	− 1	1	95.6782	90.8676	4.8105
8	16	Center	0	0	0	0	115.162	114.726	0.4362
6	17	Axial	− 2	0	0	0	66.5967	76.8237	− 10.227
5	18	Factorial	1	− 1	1	− 1	89.2802	88.9773	0.3029
17	19 2	Axial	0	0	2	0	95.9690	92.7216	3.2474
11	20	Factorial	1	− 1	− 1	1	84.0456	80.0105	4.0350
9	21	Factorial	− 1	1	1	− 1	91.316	90.2375	1.0784
25	22	Axial	0	0	− 2	0	57.2906	70.6196	− 13.329
22	23	Axial	0	2	0	0	86.3721	86.9052	− 0.5331
26	24	Factorial	1	1	1	1	93.6425	94.4059	− 0.7633
30	25	Axial	0	0	0	2	97.4231	100.912	− 3.4897
2	26	Factorial	− 1	1	1	1	101.203	102.282	− 1.0784
12	27	Factorial	1	− 1	1	1	79.1017	80.0832	− 0.9815
13	28	Factorial	− 1	− 1	1	1	84.9180	81.8523	3.0656
21	29	Factorial	− 1	1	− 1	− 1	74.1579	68.2083	5.9495
16	30	Axial	0	0	0	− 2	80.5558	87.1476	− 6.5918

Variables	Code	Coded and actual levels							
		− 2	− 1	0	1	2			
Glucose	*X*_1_	8	16	24	32	40			
(NH_4_)_2_SO_4_	*X*_2_	0.4	0.8	1.2	1.6	2			
Rock phosphate	*X*_3_	12.5	25	37.5	50	62.5			
NaCl	*X*_4_	0.4	0.8	1.2	1.6	2			

### Multiple regression analysis and ANOVA

To evaluate and interpret the CCD experimental data findings, arithmetic operators of multiple regression statistical analysis and ANOVA computations, an integral approach to assessing the relevance and appropriateness of the quadratic regression model, were used as tabulated in Table 4. The determination coefficient *R*^2^-value of the current model is 0.91, in which 91 percent of the variation in ACP output was ascribed to independent factors, and only 9% of the overall discrepancy generated by variables could not be explained by the model and could not explain ACP activities. The model is characterized by an adjusted *R*^2^ value of 0.82, which reflected a significant correlation between theoretical and experimental results and the model's high relevance, and an experiment's accuracy and dependability are indicated by the low coefficient of variation (CV = 7.47%) as well. An appropriate signal-to-noise ratio was determined by the precision value of 10.354. Fisher's *F*-test of 10.85 and the low probability *p* of 0.000001976 indicate that the chosen model is very meaningful, as evidenced by the ratio of mean square regression and mean square residual. The smallest standard deviations may be found using quadratic regression (6.69). According to the results reported in Table 4, the positive coefficients for *X*_*2*_, *X*_*3*_, *X*_*4*_, *X*_*1*_*X*_*3*_*, X*_*2*_*X*_*3*_, and *X*_*2*_*X*_*4*_ show that these variables' linear and mutual effects boost the throughput of the ACP, as it was apparent from their *F*-values, *p*-values, *t*-values, confidence levels, and contribution percent. Contrarily, the antagonistic was apparent in the linear and mutual interaction and quadratic effects of *X*_*1*_*, X*_*1*_*X*_*2*_, *X*_*1*_*X*_*4*_, *X*_*3*_*X*_*4*_, *X*_*1*_^*2*^, *X*_*2*_^*2*^, *X*_*3*_^*2*^, and *X*_*4*_^*2*^, respectively. Based on the negative sign of a coefficient and arithmetic indications for their significance degrees, they had not significantly contributed to the enhancement of ACP production by the strain under study. The second-order polynomial model is suitable for optimization and inference of nontrivial events since it is reasonably flexible and can accurately describe curvature and interactions.

**Table 4:** Analysis of variance for the response surface of ACP production by *B. haynesii* strain ACP1 obtained by RCCD.

Term	Coefficient Estimate	Mean square	*t*-Stat	*F*-value	*p*-value	Confidence level (%)	Contribution %
Intercept	114.726	486.42	41.97	10.85	5.705E^−17^		
*X*_1_	− 1.0663	27.28	− 0.78	0.608	0.447	55.26	1.892
*X*_2_	1.9630	92.48	1.43	2.063	0.1714	82.85	3.483
*X*_3_	5.5254	732.74	4.04	16.34	0.00106	99.89	9.804
*X*_4_	3.4413	284.22	2.51	6.341	0.02363	97.63	6.106
*X*_1_*X_2_	− 1.5267	37.29	− 0.91	0.832	0.37607	62.39	2.709
*X*_1_*X_3_	0.3635	2.114	0.217	0.047	0.83098	16.90	0.645
*X*_1_*X_4_	− 1.7085	46.70	− 1.02	1.042	0.32352	67.64	3.031
*X*_2_*X_3_	3.1989	163.73	1.911	3.653	0.07526	92.47	5.676
*X*_2_*X_4_	3.5261	198.93	2.106	4.438	0.05237	94.76	6.256
*X*_3_*X_4_	− 2.6536	112.67	− 1.585	2.513	0.13369	86.63	4.708
*X*_1_*X_1_	− 10.008	2747.7	− 7.829	61.30	1.12E^−06^	99.999	17.75
*X*_2_*X_2_	− 7.9368	1727.8	− 6.208	38.55	1.67E^−05^	99.998	14.08
*X*_3_*X_3_	− 8.2640	1873.1	− 6.464	41.79	1.07E^−05^	99.998	14.66
*X*_4_*X_4_	− 5.1740	734.29	− 4.047	16.383	0.00105	99.89	9.180

	df	SS	MS	*F*	Significance *F*		
**Regression**	14	6809	486	10.85	1.97634E^−05^	**Std. Dev**	6.69
**Residual**	15	672	44			**Mean**	89.62
**Total**	29	7482				**C.V. %**	7.47
***R***^*2*^	0.91					**PRESS**	3868
**Adj.** ***R***^*2*^	0.82						
**Lack of Fit**	10						

“Std. Dev. is the standard deviation, the coefficient of determination (*R*^*2*^), Adj *R*^*2*^ is the adjusted-*R*^*2*^, and PRESS is the prediction error sum of squares, C.V is the Coefficient of variation.”

The regression coefficients were computed to fit the second-order polynomial equation (Table 4). By *B. haynesii* strain ACP1, ACP throughput (*Y*) is expressed as a regression equation:

$$\begin{aligned} Y_{activity } & = 114.7 - 1.06X_{1} + 1.96X_{2} + 5.52X_{3} + 3.34X_{4} - 1.52X_{1} X_{2} \\ & \quad + 0.36X_{1} X_{3} - 1.7X_{1} X_{4} + 3.19X_{2} X_{3} + 3.52X_{2} X_{4} \\ & \quad - 5.65X_{3} X_{4} - 10X_{1}^{2} - 7.93X_{2}^{2} - 8.26X_{3}^{2} - 5.17X_{4}^{2} \\ \end{aligned}$$

where *Y* is the predicted response (ACP activity), and *X*_*1*_, *X*_*2*_, *X*_*3*,_ and *X*_*4*_ are the coded levels of the independent variables of glucose, (NH_4_)_2_SO_4_, RP, and NaCl, respectively.

### Model adequacy checking

**Figure 4:**
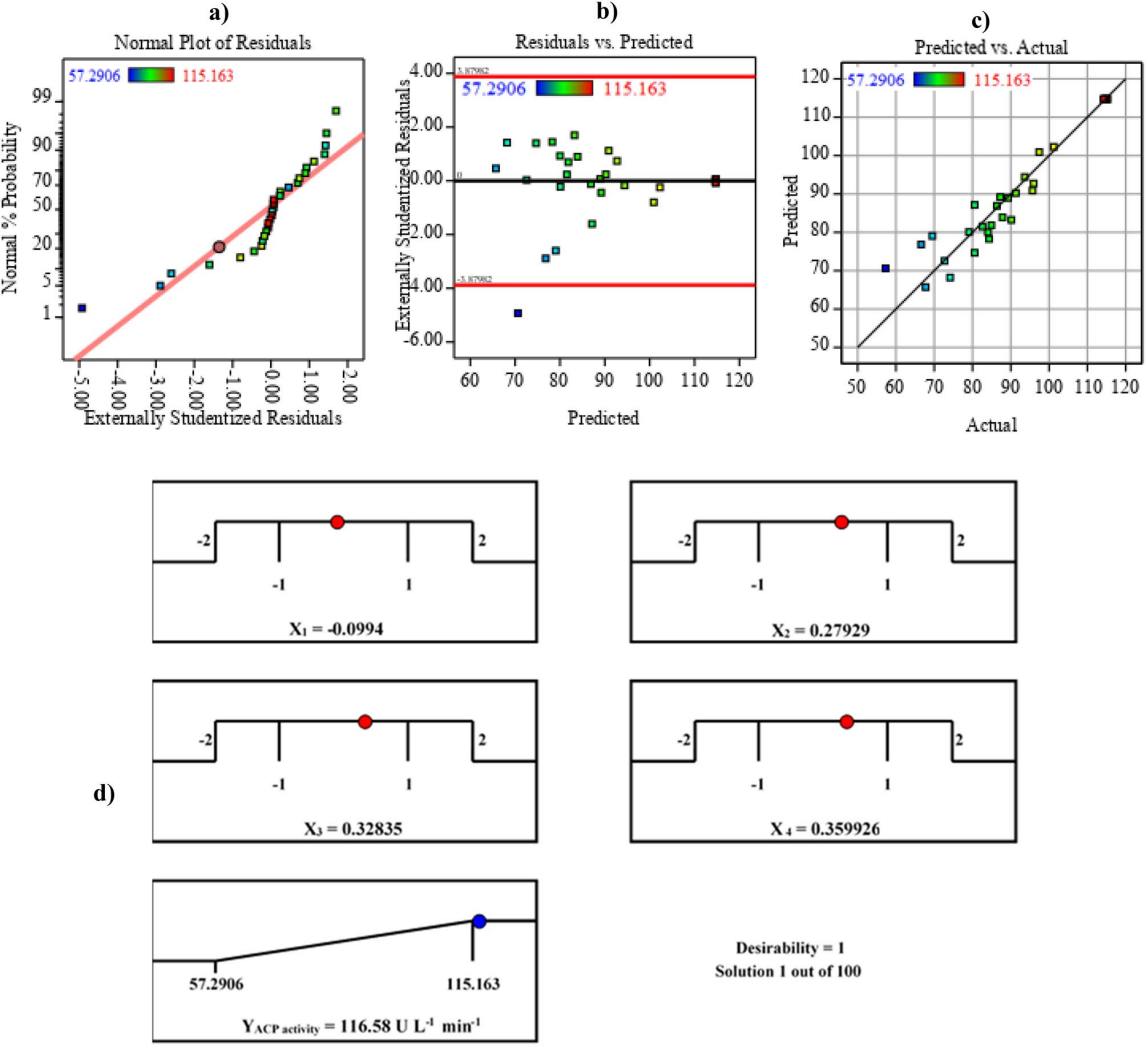
Model adequacy checking of Rotatable central composite design, **(a)** normal probability plot of the residuals, **(b)** externally studentized residuals versus predicted acid phosphatase production, **(c)** plot of predicted versus actual acid phosphatase production, and **(d)** the optimization plot displays the desirability function and the optimum predicted values for the maximum acid phosphatase production.

The data points in (Fig. 4a) are clustered tightly along the straight line, and the values that deviated from the overall mean and decreased equally on both sides of the central peak are undesirable^19^. The residuals are shown against the predicted response in Fig. 4b, allowing the assumption of constant variance to be confirmed. There was a random distribution of points in the experimental runs RSM's models were found to be adequate and satisfy the constant variance assumption because all values were within a 0.4 to − 0.6 range. Additionally, (Fig. 4c) shows that the data points are evenly distributed along a 45° line. This may be used to determine if the model can not predict particular values. Finally, it's worth noting that all of the data points can be retrieved, proving the model's accuracy.

### Optimization using the desirability function

Figure 4d depicts the optimization plot with the highest predicted values and the desirability function for *B. haynesii* strain ACP1 producing the ACP. *B. haynesii* strain ACP1 recorded the highest predicted value of ACP (116.5 U L^−1^) in the presence of g L^−1^: glucose (23.2), (NH_4_)_2_SO_4_ (1.3117), RP (41.6), and NaCl (1.3439) without pH adjustment under cultivation of 50 °C and 200 rpm for 24 h. Laboratory validation was utilized to corroborate the theoretical data results of the optimization process. The verification experiment revealed that the experimental findings and their predicted values are silently in deep agreement, implying that the desirability function efficiently computed the ideal predicted conditions for ACP production by *B. haynesii* with approximately 99.8 percent accuracy.

### Contour and three-dimensional (3D) plots

**Figure 5:**
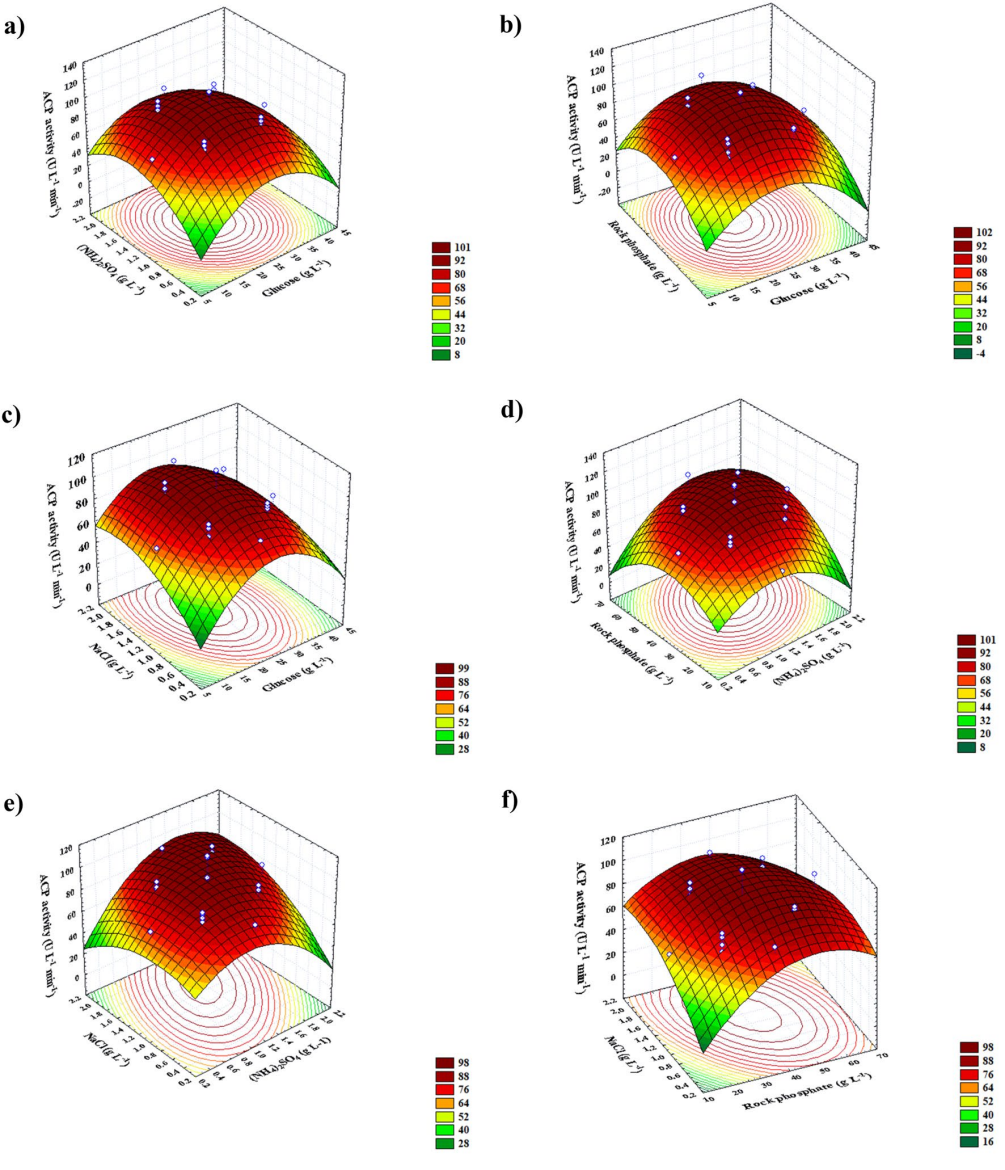
3D response surface representing acid phosphatase activity yield (U L^−1^ min^−1^) from *B. haynesii* strain ACP1 as affected by culture conditions, **(a)** interaction between (NH_4_)_2_SO_4_ and glucose, **(b)** interaction between rock phosphate and glucose, **(c)** interaction between NaCl and glucose, **(d)** interaction between rock phosphate and (NH_4_)_2_SO_4_, **(e)** interaction between NaCl and (NH_4_)_2_SO_4_, and **(f)** interaction between NaCl and rock phosphate.

As a result of charting response (ACP activity) on the *z*-axis against two independent factors while keeping other variables at zero levels (center points), three-dimensional plots were constructed for the significant pair-wise combinations of the four variables (*X*_*1*_*X*_*2*_, *X*_*1*_*X*_*3*_, *X*_*1*_*X*_*4*_, *X*_*2*_*X*_*3*_, *X*_*2*_*X*_*4*,_ and *X*_*3*_*X*_*4*_), as plotted in Fig. 5. A 3D surface map showing the simultaneous influence of glucose and (NH_4_)_2_SO_4_ on ACP production was created (Fig. 5a). Around the glucose center, ACP activity was at its peak, while outside of this area, ACP yield was minimal. In addition, as (NH_4_)_2_SO_4_ concentrations were increased, ACP activity increased until it reached its optimum around the center points of (NH_4_)_2_SO_4_, but the greater level supporting low ACP activity was noted. The greatest predicted ACP activity of 114.88 U L^−1^ was found at the optimal predicted glucose (23.2 g L^−1^) and (NH_4_)_2_SO_4_ (1.3117 g L^−1^) concentrations at RP and NaCl concentrations of 37.5 and 1.2 g L^−1^, respectively. Moreover, according to Fig. 5b, RP levels (*X*_*3*_) at lower and higher levels are associated with a reduction in ACP activity. However, the highest ACP activity (115.67 U L^−1^) is located close to both glucose (*X*_*1*_) and RP's central point (*X*_*3*_). This emphasizes the roles of glucose (*X*_*1*_) and RP (*X*_*3*_) in ACP biosynthesis. A similar trend of ACP efficiency was observed for the other pairwise combination of variables under consideration. In Fig. 5c, ACP production reached its peak (115.36 U L^−1^) when NaCl (*X*_*4*_) was increased to 1.3 g L^−1^ and at the center point of glucose (*X*_*1*_), while the two other variable variables remained at zero. It was noticed that when using (NH_4_)_2_SO_4_ (*X*_*2*_) and RP (*X*_*3*_) at middle concentrations, as indicated in Fig. 5d, the maximum throughput of ACP was reached (115.95 U L^−1^); any further increase or decrease was followed with a loss in the productivity of the ACP. Whilst maintaining glucose (*X*_*1*_) and RP (*X*_*3*_) at their zero levels, raising NaCl concentration slightly beyond its center point enhanced ACP productiveness (115.63 U L^−1^) when (NH_4_)_2_SO_4_ was present near its mid-point, but increasing both concentrations above their recorded point led to a drop in ACP yield as illustrated in Fig. 5e. An analysis of the Fig. 5f plot indicates that lower and higher concentrations of RP (*X*_*3*_) and NaCl (*X*_*4*_) sustain relatively modest yields of ACP production. However, the highest ACP production (115.97 U L^−1^) is located near the NaCl and RP center points. This means that there is no substantial association between the two variables and that they did not have much of an impact on boosting the output of ACP as a whole. There is no doubt about it: the presence of RP is crucial to enhancing ACP throughput as shown in Fig. 5b,d,f.

### Scale-up fermentation strategies for ACP production by *B. haynesii *strain ACP1

#### Cell growth kinetics and ACP production in shake-flask under batch conditions

Researchers have a common assumption that the best medium chosen from shake flask data will be the best medium in a large-scale stirred tank^20^. In the current investigation, the production of extracellular ACP was monitored throughout the growth of *B. haynesii* strain ACP1 on an optimized medium in a shake-flask under standard cultivation conditions to find out what relationship there is between the culture's specific growth rate and the rate of ACP production using a cell growth kinetics strategies. Table 5 shows how different cultivation strategies impact *B. haynesii* strain ACP1's cell growth kinetics and ACP production characteristics. The growth rate of *B. haynesii* strain ACP1 biomass can be expressed kinetically as^21^:

$$\frac{dx}{{dt}} = \mu x$$

where “x” is biomass concentration (g L^−1^), “µ” is specific growth rate (h^−1^), and “t” is time (h).

$$\mu = \frac{{\mu_{max} s}}{{K_{s} + S}}$$

where “μ” is the specific growth rate (h^−1^), “S” is substrate concentration (g L^−1^), “K_S_” is the Monod constant (g L^−1^), and “μ_max_” is the maximum specific growth rate (h^−1^).

**Table 5:** Kinetic parameters of cell growth and ACP production by *B. haynesii* strain ACP1 as affected by different cultivation strategies.

Parameters	Shake flask cultivation	Uncontrollable pH batch cultivation	Controllable pH batch cultivation
**Growth parameters**			
*X*_max._ (g L^−1^)	9.311	3.101	2.358
$\mu$ (h^−1^)	1.17	0.07	0.05
$\mu_{max}$ (h^−1^)	0.546	0.506	0.251
$\frac{dX}{{dt}}$ (g L^−1^ h^−1^)	0.452	0.102	0.083
**Production parameters**			
*P*_max_ (U L^−1^)	111.674	207.645	114.583
*P*_max.specific_ (U g^−1^)	103.266	222.769	159.755
*P*_max.time_ (h)	24	28	26
*Q*_*p*_ (U L^−1^ h^−1^)	2.6658	5.01664	3.41052
− *Q*_*s*_ (g L^−1^ h^−1^)	1.03	0.7	0.3
**Yield coefficient parameters**			
*Y*_*p/s*_ (U g^−1^)	4.3	8.21	5.74
*Y*_*p/x*_ (U g^−1^)	9.81	25.2	23.1
*Y*_*x/s*_ (g g^−1^)	0.31	0.23	0.22
Overall cultivation time (h)	30	22	32

*X*_max._, maximal cell dry weight; $\frac{dX}{{dt}}$, cell growth rate; µ, specific growth rate; *P*_max_, maximal ACP production; *P*_max specific_, specific productivity; *Q*_*p*_, ACP production rate; *Q*_*s*_*,* substrate consumption rate; *Y*_*p/x*_, U g^−1^ of ACP produced per g biomass; *Y*_*p/s*_ U g^−1^ of ACP produced per g substrate consumed, and *Y*_*x/s*_ g g^-1^, of biomass produced per g substrate consumed.

**Figure 6:**
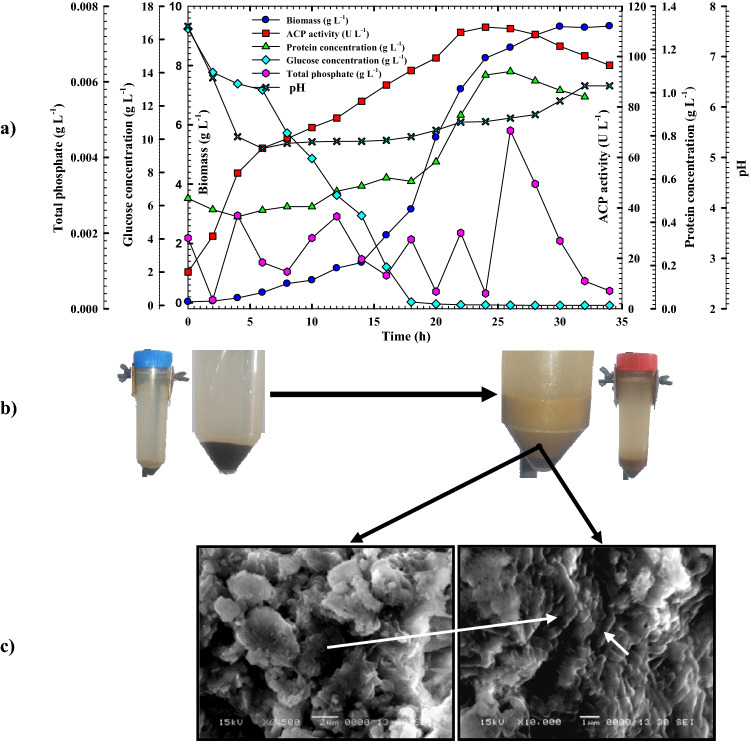
Monitoring of *B. haynesii* strain ACP1growth and acid phosphatase productivity in **(a)** a shake-flask scale cultivation condition, **(b)** rock phosphate leftover sample at the lag phase, and rock phosphate leftover sample at the end of the incubation time showing the solubilization process of rock phosphate, and **(c)** morphological characterization of RP surface illustrated the attached bacterial cells.

As evident from Fig. 6a, *B. haynesii* strain ACP1 grew normally like other *Bacillus* sp., and ACP was produced concurrently with cell growth. Following a lag period, cells exhibited exponential growth with a growth rate of 0.452 (g L^−1^ h^−1^) and a specific growth rate (*µ*) of 0.137 h^−1^. In a 28 h cultivation period, the maximum biomass yield (9.31 g L^−1^) was reached, with an apparent yield coefficient *Y*_*x/s*_ (0.31 g g^−1^), which was exceeded, causing the cells to enter the stationary phase. It was found that the ACP responsible genes were expressed gradually from the beginning of the cultivation for the production of ACP until its crest (111.6 U L^−1^) at 24 h through a production rate (*Q*_*p*_) of 2.66 U L^−1^ h^−1^. Because the cells grow at the beginning of the cultivation period more slowly, they have more time to produce ACP. The specific productivity *P*_*max specific*_, and yield coefficient *Y*_*p/s*_ for ACP were recorded as (103.2 and 4.3 U g^−1^, respectively). The same remarks were noticed in the case of the protein content trend, the protein content pattern reached its apex (1.08 g L^−1^) at the same time as the ACP production peak (i.e. at 24 h); over time, ACP activity and protein content began to decrease. Cell growth and proliferation as well as ACP's critical function for transport and metabolism of phosphate caused the glucose concentration to drop from its starting concentration of 16.64 to just 0.024 g L^−1^, with an average consumption rate of 1.03 g L^−1^ h^−1^. The tracking of phosphate content pattern revealed that the liberation of inorganic P into the culture media as a result of the bio-solubilization of RP by *B. haynesii* strain ACP1 through organic acid and ACP production resulted in the formation of inorganic P_i_, which was subsequently taken up as a nutrient for bacterial growth. For that, it is clear from Fig. 6a, that the inorganic phosphate content has a fluctuating trend throughout the fermentation period. The up and down peak of phosphate content pattern referred to the liberation (formation) of inorganic phosphate from RP (upward peak) and the consumption of the inorganic phosphate by bacterial cells (downward peak). As shown in Fig. 6a,b, at the beginning of the cultivation process during a lag phase, there is no obvious RP-solubilization accrue as a result of the slow growth of bacterial cells at this stage. On the other hand, after 26 h from incubation time, it was observed that complete solubilization of organic phosphate was achieved with the highest peak of inorganic phosphate (0.0047 g L^−1^) formation at that time; that peak was in between cell growth and ACP production peaks As seen in Fig. 6c, the bacterial cells firmly adhered to the surface of the RP particles. The solubilization process was associated with a reduction in pH, suggesting the participation of [H^+^] in the solubilization mechanism, as described by Kang et al.^22^. The following equation defines the solubilization of PR under acidic conditions:

$${\text{Ca}}_{3} \left( {{\text{PO}}_{4} } \right)_{2} + 4{\text{H}}^{ + } \leftrightarrow 3{\text{Ca}}^{2 + } + 2{\text{PO}}_{4}^{3 - } + 4{\text{H}}^{ + } \leftrightarrow 3{\text{Ca}}^{2 + } + 2{\text{HPO}}_{4}^{2 - } + 2H^{ + } \leftrightarrow 3{\text{Ca}}^{2 + } + 2{\text{H}}_{2} {\text{PO}}_{4}^{ - }$$

Because of this, the pH of *B. haynesii* strain ACP1 cultural media was progressively dropped from 7.5 to 5.18 during the lag phase, then steadily stable throughout the log phase (from 6 to 20 h) with a pH range of 5.28–5.41, and then gradually increased again to pH 6.42 at the end of the cultivation time as illustrated in Fig. 6a. Gradually, bacterial growth, ACP productivity, and protein content increased along with the pH shift to achieve their peak. Due to bacterial growth being influenced by pH and/or gene expression for enzyme production being controlled by pH as described by Qureshi et al.^23^. Additionally, an early study by Butler et al.^24^ found that the ACP activity of batch-grown cells of a *Citrobacter* sp. rose about thrice throughout exponential growth; this finding was analogous to the current study. Therefore, it appears that the *B. haynesii* strain ACP1 has a higher probability of serving as an agent for converting insoluble RP into soluble forms.

#### Cell growth kinetics and ACP production in the bioreactor under uncontrolled pH of batch fermentation condition

**Figure 7:**
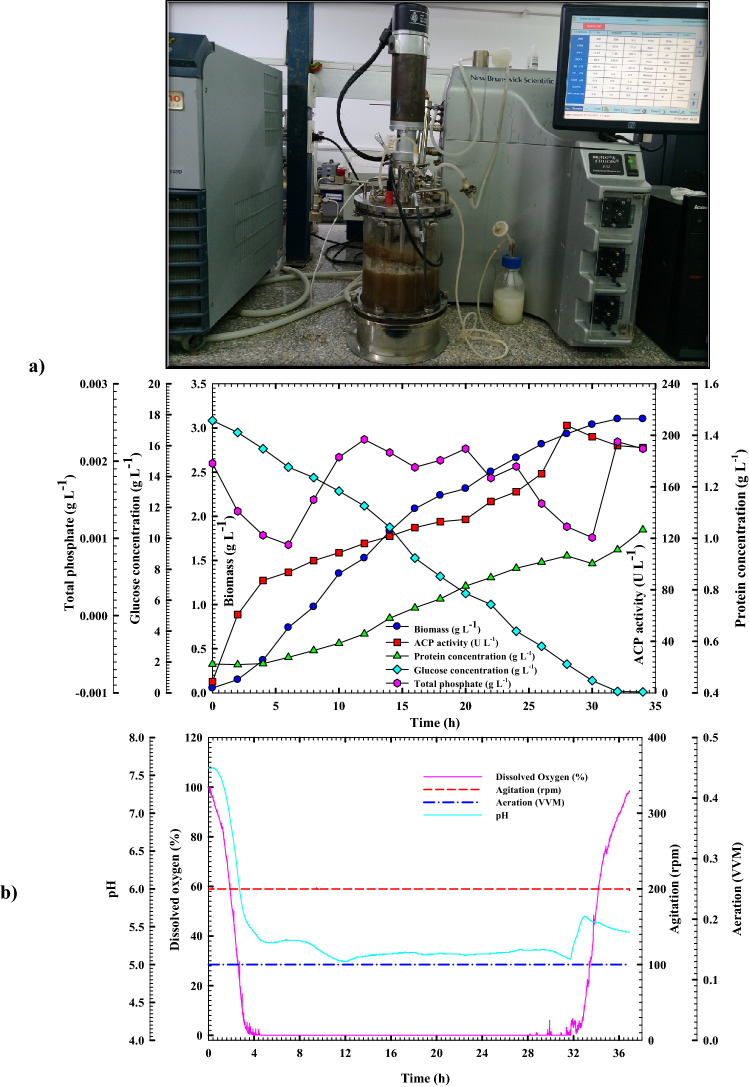
(**a)** Monitoring of *B. haynesii* strain ACP1 growth and acid phosphatase productivity in a 7 L stirred-tank bioreactor under uncontrolled pH conditions, and **(b)** online data (dissolved oxygen percentage, agitation, aeration, and pH) as a function of time during batch fermentation in the bioreactor under uncontrolled pH conditions.

The present study revealed that using the bioreactor cultivation system, the highest improved and boosted ACP production rate was achieved although ACP's time-to-production has become lengthier than those of its shake flask counterpart (Fig. 7a). Whereas the maximum ACP production for the highest ACP specific productivity (222.76 U g^−1^) with production rate *Q*_*p*_ (5.01 U L^−1^ h^−1^) was recorded as 207.6 U L^−1^ time by reaching such a point, the production curve began to flatten off. The obtained results exceeded those reported in the shake flask case by 1.86 times and by ~ 2.15 times in terms of specific productivity as illustrated in Table 5. These results were followed by significant increases in ACP yield coefficients *Y*_*p/x*_ and *Y*_*p*/s_ of 25.2 and 8.21 U g^−1^ respectively, by a factor of 2.56, and 1.9, respectively, more than those achieved at shake flask mode. Moreover, the ACP production increment was associated with a gradually increasing protein content trend; the protein concentration plummeted fast at 28 h after overstepping its peak point (0.93 g L^−1^). *B. haynesii* strain ACP1, on the other hand, did not grow as well in stirred tank bioreactors as it did in a shake flask. With a growth rate of 0.102 g L^−1^ h^−1^ and a specific growth rate (*µ*) of 0.07 h^−1^, the biomass production peaked (*X*_*max*_) at 3.1 g L^–1^ after 30 h, which is about 34.4 percent lower than what was achieved using shake-flask cultivation mode. A *B. haynesii* strain ACP1 yield coefficient *Y*_*x/s*_ of 0.23 g g^−1^ was gained under the above cultivation conditions, which is lesser than what was obtained through shake-flask cultivation mode by 25%. The glucose consumption pattern was followed throughout the cultivation process; it was found that there is an inverse proportion interrelationship between glucose concentration and *B. haynesii* strain ACP1 cell growth and ACP production. Substrate consumption rose along with cell growth and ACP production with a consumption rate (*Q*_*s*_) of 0.7 g L^−1^ h^−1^ (Table 5). The glucose concentration decreased from its starting concentration of 17.6 g L^−1^ to its lowest concentration of 0.05 g L^−1^ at the end of the cultivation period. When compared to the shake-flask cultivation system, it was discovered that the consumption rate of glucose was accomplished slowly during the cultivation period, which was approximately 32% lower than what was observed in its counterpart. Furthermore, the glucose concentration had fully depleted in the shake-flask cultivation system after 26 h, but in bioreactor cultivation, the concentration of glucose depleted to 0.05 g L^−1^ at the end of the cultivation period; here, we are emphasizing the previous findings. In addition, the pH of the culture media followed the same trend as the shaking flask counterpart. Whereas, at the early log phase after 12 h of incubation, pH values dropped from 7.5 to 5.09, then remained stable until the end of the culture period with pH values in the range of 5.12–5.18. Fermentation kinetics might be drastically altered by a change in oxygen availability, according to Walker et al.^25^. It was observed that the highest ACP production was achieved when a tiny volume of air was supplied to the culture media of the bioreactor system at 0.125 VVM rather than 0.5 or 0.25 VVM. The present findings are consistent with Hallett et al.^26^, who documented that anaerobiosis boosted phosphatase production. Due to the active growing bacterial cells in the early exponential phase (4.0 h), the DO percentage dropped quickly to reach 0.5 percent. After this point, it remained constant at zero percent until the fermentation process was completed as illustrated in Fig. 7b. As a consequence of bio-solubilization of RP, the phosphate content fluctuating trend was observed throughout the cultivation time, although the solubilization process did not occur as efficiently as it did in the shake-flask cultivation system, however, the production of ACP improved more than those obtained in shake-flask. A *Bacillus* sp. isolated from forest soil in Gunung Salak National Park's Gunung Salak National Park displayed low activities between 0.02 and 1.01 U on media containing para-nitrophenyl phosphate solution as artificial organic phosphate (P_o_), according to a study done by Rahmansyah and Sudiana, 2010^27^. Although the strain ACP1 of *B. haynesii* that was used in this study produced a large amount of ACP, with an activity of 207.6 U in RP-based media. Meanwhile, *Piriformospora indica* demonstrated ACP activity with 76 U L^−1^ in RP-containing media, which was 2.73 times lower than the activity seen in the present investigation^5^. Additionally, Chen and Liu^17^ discovered in 2019 that a *Pantoea* sp. S32 isolated from an alfalfa rhizosphere in a heavy metal-contaminated reclamation area in Shanxi Province, China was capable of producing ACP with a maximum activity of 69.4 U L^−1^, which was 2.98 times lower than the activity obtained in the current study. Furthermore, the optimal ACP production of *P. aeruginosa* CH01 (18.4 U L^−1^), was attained at 96 h, which was 11.25 times less than the maximal activity obtained by the strain under study^11^. On the other hand, ACP was produced in large quantities by *Klebsiella* and *Morganella*, as well as *Providencia stuartii*. For the *Klebsiella* species, production varied from 11.63 to 18.66 U L^−1^ min^−1^, which was 17.79 to 11.09 times less than the maximal activity obtained by the strain under study, whereas for *Morganella morganii* and *Providencia stuartii*, production ranged from 170 to 675 and 1300 to 1686 U L^−1^ min^−1^, respectively^28^.

#### Cell growth kinetics and ACP production in the bioreactor under controlled pH of batch fermentation condition

**Figure 8:**
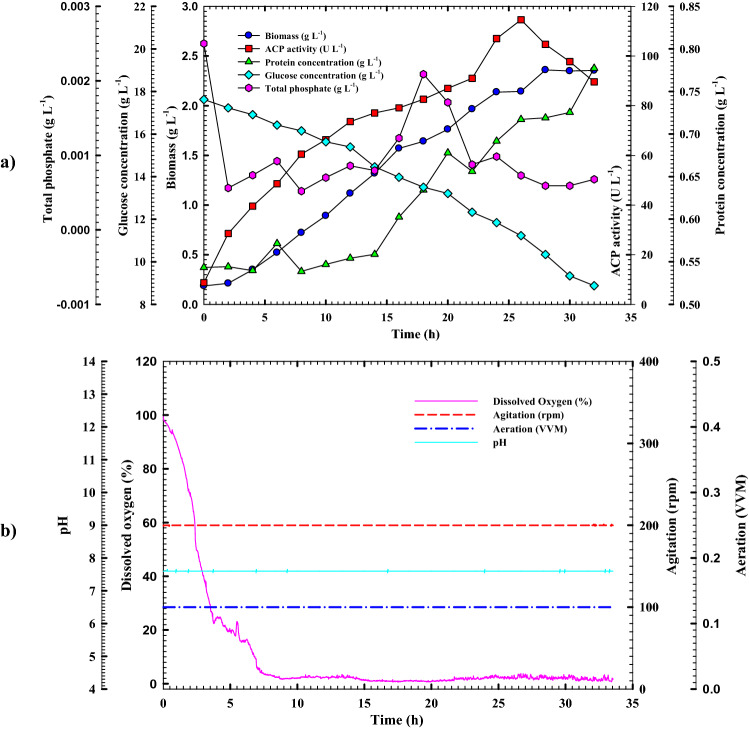
**(a)** Monitoring of *B. haynesii* strain ACP1 growth and ACP productivity in a 7 L stirred-tank bioreactor under controlled pH conditions, and **(b)** online data (DO, agitation, aeration, and pH) as a function of time during batch fermentation.

Using a bioreactor cultivation system, *B. haynesii* strain ACP1 was forced to grow at a constant pH of 7.5 to investigate the impact of constant pH value on the growth and productivity characteristics. All of the patterns in Fig. 8a are analogous to those observed in shake-flask culture. Without a substantial phase lag, the bacterial cells grew exponentially over time. The increase in cell growth was detected during a 4.0 h cultivation period with a growth rate of 0.083 g L^1^ h^1^ till it reached its peak (*X*_*max*_ = 2.35 g h L^−1^) at 28 h, which was lower than those obtained from uncontrolled pH batch cultivation, and shake-flask cultivation systems by 24.1 and 75.2%, respectively. As a result, yield coefficient *Y*_*x/s*_ of 0.22 g g^−1^ was recorded, which was lesser than those obtained from the shake flask system by 29%. Moreover, the specific growth rate (*µ*) of 0.05 h^−1^ was documented under such conditions, which was about 63.5, and 28.5% less than those obtained from shake-flask and uncontrolled pH batch cultivation, respectively (Table 5). The trends of volumetric enzyme productivity and protein content paralleled those of cell growth. Furthermore, ACP volumetric productivity with a production rate (*Q*_*p*_) of 3.41 rose steadily during the fermentation time, peaking at 26 h of 114.5 U L^–1^. Although it shifted to become earlier than those obtained uncontrolled pH system by 2.0 h, it was shown that the volumetric productivity of ACP decreased by 44.8 percent as compared to uncontrolled pH batch culture, but it showed greater activity than those produced in the shake-flask method by 2.6%. The yield coefficient *Y*_*p/x*_ (23.1 U g^–1^) is roughly 0.91 times lower than that of the culture with uncontrolled pH batching but 2.35 times greater than that of shaking flask cultivation. The same observation was recorded regarding yield coefficient *Y*_*p/*s_ of 5.74 U g^−1^, which decreased by 30, and 33.4 percent than what was achieved through uncontrolled pH batch and shake-flask cultivation mode, respectively. After 32 h of fermentation process, the glucose concentration dropped from 17.6 to 8.8 g L^−1^ with an average consumption rate of 0.3 g L^−1^ h^−1^ due to the correlation between substrate consumption and growth and enzyme production patterns. Additionally, it was noticed that the consumption of DO trend under controlled pH conditions was shifted to become more lately than those observed in uncontrolled pH cultivation system **(**Fig. 8b). The phosphate content pattern exhibited the same fluctuating trend throughout the incubation period as reported in the uncontrolled pH cultivation system. As shown in Fig. 8a, the highest peak for phosphate content (0.002 g L^−1^) was achieved after 18 h of cultivation time. Then a gradual decline in phosphate concentration trend was observed until the end of incubation time. However, unlike in the shake flask, the solubilization process was not as efficient as in the uncontrolled pH culture system. Overall the current study, unfavorable findings were achieved with bacterial cells growing and surviving under controlled pH conditions because a constant pH was detrimental to their growth and survival. Hence, the *B. haynesii* strain ACP1 could no longer produce ACP effectively. As a result, the uncontrolled pH culture condition is the most appropriate and preferred setting for promoting and boosting ACP productivity. The novel formulation media was developed using low-cost and straightforward constituents for solubilizing RP in accordance with ACP production through current study. ACP production from *B. haynesii* strain ACP1 was optimized sequentially, starting with PBD (64 U L^−1^), moving on to RCCD (111.6 U L^−1^), and finally to uncontrolled pH batch culture method designs (207.6 U L^−1^). It's difficult to compare results to others because of the lack of relevant literature. By using RP formulated medium, this work is considered the first to report on the production, statistical optimization, and scale-up production of extracellular ACP from *B. haynesii* strain ACP1 in benchtop bioreactor scale.

### Morphological structure of RP

**Figure 9:**
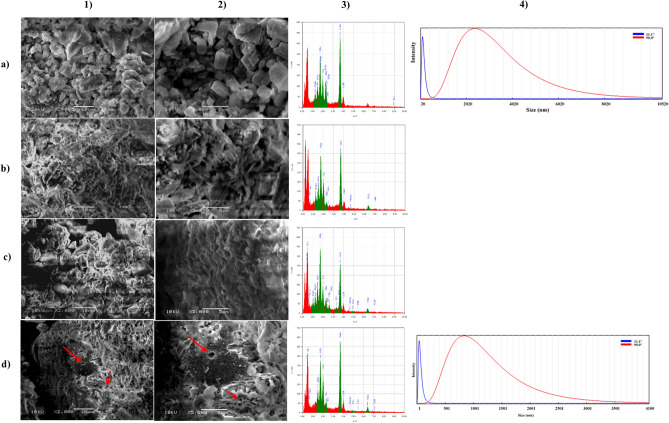
Scanning electron microscopy (SEM) micrograph of the rock phosphate leftover samples, (**a)** at zero incubation time, (**b)** after 12 h of incubation time, (**c)** after 24 h of incubation time, and (**d)** after 32 h of incubation time at a magnification **1)** 2000 x, and **2)** 5000 × with 15 kV, **3)** EDX analysis chart, and **4)** particle size distributions analysis graph.

Figure 9 clearly shows that the RP leftover particles have undergone a solubilization process by *B. haynesii* strain ACP1 throughout the fermentation process, which resulted in noticeable alterations in the morphology, structure, and elemental constitutes of the collected residues samples of RP. SEM micrograph (Fig. 9a1,a2) illustrated the morphology of RP particles at zero incubation time at magnifications 2000 × and 5000x. It can be noticed that the RP particles had a rough surface, a compact structure, a high degree of crystallinity, and a larger number of edges and corners, which is consistent with Maharana et al.^29^ findings. Moreover, the existence of Ca, P, Si, Mg, Al, S, Ti, and traces of other elements is demonstrated by EDX analysis as shown in Fig. 9a3. The three absorption peaks corresponding to Ca (AT% of 52.52 and mass% of 54.89), Si (AT% of 16.99 and mass% of 12.44), and P (AT% of 10.93 and mass% of 8.83) are the most abundant in the sample. Thus, the Ca:P weight ratio of the RP hydroxyapatite was 4.805. In addition, Bachouâ et al.^30^ observed irregular forms of Tunisian rock particles alongside the presence of Ca, P, O, Si, C, and F, which are different in metals content from that present in Egyptian RP particles and exploited in the current investigation.

On another side, after 12 h from fermentation time, as shown in Fig. 9b1,b2, the bacteria formed a rugged structure on the RP particle's surface. As a result of bacterial growth, RP morphology has altered, with particles taking on looser structure, irregular shapes, and polygonal shapes vanishing with a blurring of the surface boundaries of the granules. Meanwhile, EDX analysis revealed that the Fe appeared in small traces together with S, Al, and Mg, accompanied by the disappearance of Ti. However, Ca (AT% of 44.96 and mass% of 50.04), Si (AT% of 20.39 and mass% of 15.90), and P (AT% of 11.5 and mass% of 9.9), are still the major elements in the sample as illustrated in Fig. 9b3. It was observed that the AT% for Ca was decreased by factor 0.85 as a result of bacterial cell consumption. At the same time, about 20 and 30.3%, a noticeable elevation in AT% for Si and P was noted, respectively. This elevation is because the solubilization process carried out by *B. haynesii* strain ACP1 led to releasing such metals from RP. As a consequence, the Ca:P weight ratio of the RP hydroxyapatite was 3.909, which was lower than the obtained ratio before the fermentation process by 18.63%. In Fig. 9c1,c2, the RP particle form disappeared, and tiny pores seemed to result from bacterial cell growth and its metabolites effects. Additionally, more extra elements were detected through EDS analysis after 24 h incubation time like K, Cl, V, and Na along with the above-mentioned elements. However, the most plentiful elements were Ca (AT% of 33.59 and mass% of 38.71), Si (AT% of 21.98 and mass% of 17.74), and P (AT% of 10.32 and mass% of 9.19). It was noticed that about 25.28% and 36% reduction in Ca content (AT%) compared to that obtained at 12 h and zero incubation time, respectively. Only 10.26% less in P content (AT%) than those obtained after 12 h incubation time was observed. As a result, the Ca:P weight ratio of the RP hydroxyapatite was 3.25. About 32.26% and 16.73% reduction in Ca:P weight ratio compared to zero time and after 12 h incubation time, respectively. At the same time, the trend of Si content (AT%) is still grown-up by a factor of 1.077 and 1.29 more than those achieved at 12 h and zero incubation time, respectively (Fig. 9c3). Finally, when compared to the control sample at the end of cultivation time, the treated RP surfaces were significantly eroded by the *B. haynesii* strain ACP1, and several asymmetrical slots and pits on the RP surface were detected, as illustrated by the red arrow on the RP surface in Fig. 9d1,d2. This erosion might be due to proton assault by *B. haynesii* strain ACP1 secreted organic acid compounds and phosphate leaching, which was previously observed by Maharana et al.^29^, and Henri et al.^31^. The leftover RP particles had an irregular shape with no sharp edges that were non-uniformly distributed (monodispersed) with substantial deformation. Al, S, V, and Fe were recorded as traces by EDS analysis. Ca (AT% of 49.86 and mass% of 53.41), Si (AT% of 16.71 and mass% of 12.54), and P (AT% of 13.76 and mass% of 13.39) were identified as the highest peaks of the elements map (Fig. 9d3). The increment of Ca and P content by factors 1.48 and 1.33, respectively, than those obtained at an incubation time of 24 h, was detected as a result of the completion of the solubilization process of RP. Ca:P weight ratio of the RP hydroxyapatite was recorded as 3.62. This abrupt change in RP composition suggested that bacteria-mediated solubilization had a substantial influence.

Moreover, the particle size distributions analyzer determined the arithmetic mean particle size at multi-angle of 90 and 11.1°. The analysis demonstrated that the particle size of RP after 32 h of incubation time was 912.1 and 46.6 nm, respectively. In contrast, the particle size of RP before the fermentation process was 2554.8 and 115.2 nm, as shown in Fig. 9a4,d4. This noticeable reduction in the size of RP particles is a result of bio-solubilization and fermentation processes, which in turn aided the release and leaching of some minerals and other soluble components from the rock particle led to a reduction in the size of the particle.

### Organic acids production profile

**Figure 10:**
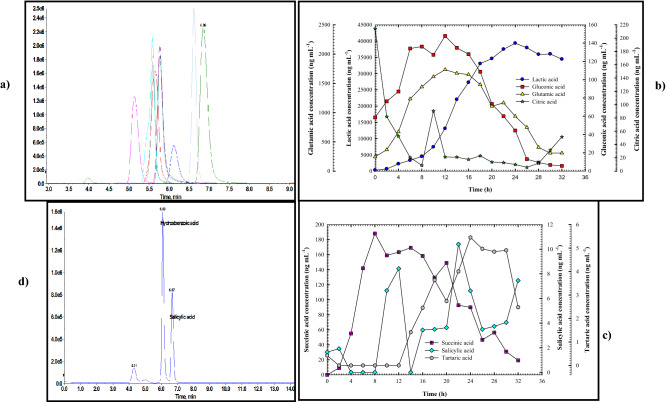
(**a)** Representative extracted negative ion MRM chromatograms of organic acids at the MQC: glutamic acid (*m/z*: 145.96/128.0, RT: 5.13 min), gluconic acid (*m/z*: 194.95/129.1, RT: 5.59 min), succinic acid (*m/z*: 116.88/72.9, RT: 5.60 min), lactic acid (*m/z*: 88.62/43.0, RT: 5.61 min), citric acid (*m/z*: 190.92/111.0, RT: 5.75 min), tartaric acid (*m/z*: 148.69/87, RT: 5.77 min), pyruvic acid (*m/z*: 86.94/43.0, RT: 6.12 min), salicylic acid (*m/z*: 136.94/93.0, RT: 6.63 min), and maleic acid (*m/z*: 114.91/71.0, RT: 6.85 min), (**b)** monitoring of lactic, gluconic, glutamic, and citric acids production, (**c)** monitoring of succinic, salicylic, and tartaric acids production throughout the shake-flask cultivation system and (**d)** representative extracted negative ion MRM chromatograms illustrating the presence of hydroxybenzoic acid alongside salicylic acid.

*B. haynesii* strain ACP1 shake-flask cultivation broth was analyzed by LC–MS/MS for organic acids detection (lactic acid; maleic acid; succinic acid; glutamic acid; citric acids; salicylic acid; gluconic acid; tartaric acid) at different intervals incubation times. Figure 10a shows the extracted negative ion multiple reaction monitoring (MRM) chromatograms of the identified organic acids as a standard. It was found that lactic acid was the most abundant (39,350 ng mL^−1^) among the eight distinct organic acids, followed by glutamic (1734.5 ng mL^−1^), succinic acids (188.10 ng mL^−1^), gluconic acid (147.8 ng mL^−1^), salicylic acid (10.4 ng mL^−1^) and tartaric was the least abundant (5.4 ng mL^−1^) alongside hydroxybenzoic acid isomer.

By tracking the production of organic acids throughout the fermentation process, it was noted that as shown in Fig. 10b, the lactic acid production was synchronous with the growth of *B. haynesii* strain ACP1 and ACP production patterns. In comparison, the lactic acid production trend was gradually increased from (272.6 ng mL^−1^) until it reached its peak (39,350 ng mL^−1^) at the same time as the ACP production peak (i.e. at 24 h), over time, lactic acid production steadily stable until the end of cultivation time. The glutamic acid and gluconic acid production had the same trend as the lactic acid production trend. In almost the same way, each kept ascending growth until both reached their pinnacle (1734.5 and 147.8 ng mL^−1^, respectively) at 12 h, the time equivalent to the end of the lag phase of *B. haynesii* strain ACP1 growth and the beginning of ACP production time and after transcending this point, a gradual decline in production of both were observed. Organic acids production peaked at the same time that the phosphate content peaked (12 h). This emphasizes organic acid's important involvement in RP solubilization, and the pH of culture broth at that time was recorded as 5.32 as displayed in Fig. 6. Contrary to mentioned, the tracking of citric acid concentration was considered as the trend of consumption throughout the fermentation process, not as a production trend. It was observed from Fig. 10b that the progressively increment of cell growth, and ACP production caused a sharp decline in the concentration of citric acid from its initial concentration of 214.2 ng mL^−1^ at zero time to 5.6 ng mL^−1^ after 26 h of incubation time, beyond this time a gradual increment in citric concentration was seen. It is noteworthy that, the tartaric acid production began during the early log phase of *B. haynesii* strain ACP1's growth (14 h) and culminated (5.4 ng mL^−1^) at 24 h of fermentation time, which coincided with the peak point of ACP production. During the first 12 h of incubation time, no production is seen (Fig. 10c). Meanwhile, a peak in succinic acid production (188.1 ng mL^−1^) was attained after 8 h of incubation time, following which the production curve began to decline progressively with fluctuation from that point on throughout the fermentation process. Due to bacterial growth, it was observed a fluctuation trend in salicylic acid production after 10 h of production. Correspondingly, it was seen the presence of hydroxybenzoic acid isomer with a molecular weight of 138 in a reasonable amount, whereas, hydroxybenzoic acid is an isomeric compound of salicylic acid and is present in the same MRM channel, but they are baseline separated in the chromatogram (Fig. 10d). Maleic acid, on the other hand, was not produced during the entire fermentation process. Ultimately, results revealed that most organic acid standards were identified in culture, and their concentration fluctuated over time. The organic acids emitted by *B. haynesii* strain ACP1 were critical in the acidification of the broth, as seen by the reduction in pH, and therefore contributed to the solubilization of RP.

It was found that through the study of Behera et al.^8^, the lactic acid was the most prolific acid among others, which was produced during the growth of *Serratia* sp., this is in line with the findings of the current study. Moreover, in *P. ostreatus* culture broth, lactic acid was the most prevalent of five different organic acids, followed by citric acid as documented by Maharana et al.^29^. Additionally, *B. liqueniformis* and *B. amyloliquefaciens* strains were discovered to produce lactic, isovaleric, isobutyric, and acetic acid mixtures. Organic acids such as glycolic, oxalic, malonic, succinic acid, and others, have been discovered among phosphate solubilizers^15^. These documented findings have emphasized the predominance of lactic acid production among different species of microorganisms. It was shown that *A. japonicas*' solubilization of RP resulted in the highest amount of gluconic acid production among other organic acids, as reported by Xiao et al.^20^, which contradicts the present findings.

### Heavy metals survey

The shake-flask cultivation broth and leftover RP samples were subjected to atomic absorption spectrometry (AAS) analysis to detect the existence of heavy metals. *B. haynesii* strain ACP1's solubilization of transition and alkali metals (Pb^+^, Fe^+^, Cd^2+^, Mg^+^, Ca^2+^) from RP is caused by the simultaneous action of the phosphatase enzyme and the production of organic acid throughout the fermentation process, reaching their highest concentrations (1.455, 38.26, 0.155, 246.45, and 709.44 mg L^−1^, respectively) after 20 h of incubation as cited in Table 6. Overstep this time led to a gradual reduction in these concentrations until the end of the fermentation period due to the metals ion requirement for bacterial cell growth. During the first 12 h of the fermentation process, the Na^+^ concentration spiked and peaked at 1825.59 mg L^−1^. Beyond this time, the Na^+^ concentration slumped. In contrast, after 28 h of incubation time, the pinnacle concentrations of K^+^, Mn^2+^, and Cu^2+^ were found to be 506.34, 4.19, and 0.29 mg L^−1^. On the other side, AAS analysis of residual RP samples revealed that the shake-flask cultivation process resulted in the solubilization of excessive amounts of Fe^+^, Cd^2+^, Mn^2+^, Li^+^, Ca^2+^, Ag^+^, and Na^+^, their concentrations had augmented by a factor of 1.36, 1.19, 5.33, 15.12, 1.49, 1.47, and 688.5 when compared to those detected in the solid sample before fermentation. According to the obtained results, Na^+^, Li^+^, and Mn^2+^ were the most detached metals from RP during the solubilization process by *B. haynesii* strain ACP1. A bioreactor with uncontrolled pH batch culture yielded higher concentrations of the metal cations liberated Cr^+^, Zn^+^, Mg^+^, Mn^2+^, Li^+^, and Na^+^ than samples taken at zero time by a factor of 2.11, 5.77, 1.97, 15.78, 44.36, and 1153.3, respectively (Table 6). The obtained results show that the liberation of heavy metals from RP particles was more efficiently achieved in the bioreactor system than in the shake-flask system, highlighting ACP's function in the liberation of metals from the RP particle. There were significant elevations in the liberation of Mn^2+^ (195.9 percent), Li^+^ (193.3 percent), and Na^+^ (67.5%) in the bioreactor fermentation process as compared to the shake-flask fermentation process.

**Table 6:** Flame atomic absorption spectrometry for heavy metals analysis of culture filtrate samples during shake-flask batch cultivation and RP leftover samples during shake-flask, and uncontrollable pH batch cultivation system.

Metals conc. (mg L^−1^)	Incubation time (h)					
	0	4	12	20	28	32
**Flame atomic absorption spectrometry for culture filtrate**						
K	158.29	227.19	365.4	484.99	**506.34**	351.74
Pb	< 0.3535	< 0.353	< 0.353	**1.455**	0.353	< 0.353
Fe	1.890	3.62	10.081	**38.26**	15.230	12.757
Cd	0.0222	0.0373	0.1276	**0.155**	0.0437	0.047
Zn	1.49	0.350	0.6989	1.375	0.874	0.2183
Mg	83.1	95.47	123.98	**246.45**	127.46	85.46
Mn	0.9	1.46	2.157	3.727	**4.19**	3.55
Ca	349.88	536.36	660.53	**709.44**	633.28	458.219
Na	611.20	1035.01	**1825.59**	1135.26	795.18	641.50
Cu	< 0.0346	< 0.0346	0.0535	0.1339	**0.293**	0.0601

Metals conc. (mg Kg^−1^)	Rock phosphate sample (control)	Shake flask batch cultivation	Uncontrollable pH batch cultivation			
**Flame atomic absorption spectrometry for RP leftover samples**						
Co	10.32	9.616	10.01			
K	9777.00	2166.00	4186.36			
Fe	105,944.4	**145,140**	43,902			
Ni	24.420	20.420	1.58			
Cd	0.968	**1.1564**	0.47			
Cr	15.29	7.0880	**32.3400**			
Zn	1255.5	95.52	**7254.54**			
Mg	28,944.400	17,454.00	**57,286.300**			
Mn	364.5	**1944.0**	**5754.00**			
Li	77.77	**1176.0**	**3450.00**			
Ca	153,629.6	**229,000**	135,272.72			
Sn	10.120	< 1.2	< 1.2			
Ag	288.8	**426**	1.236			
Na	16.60	**11,430**	**19,145.45**			
Cu	1422.22	13.90	20.81			

Significant values are in [bold].

### Thermal analysis

**Figure 11:**
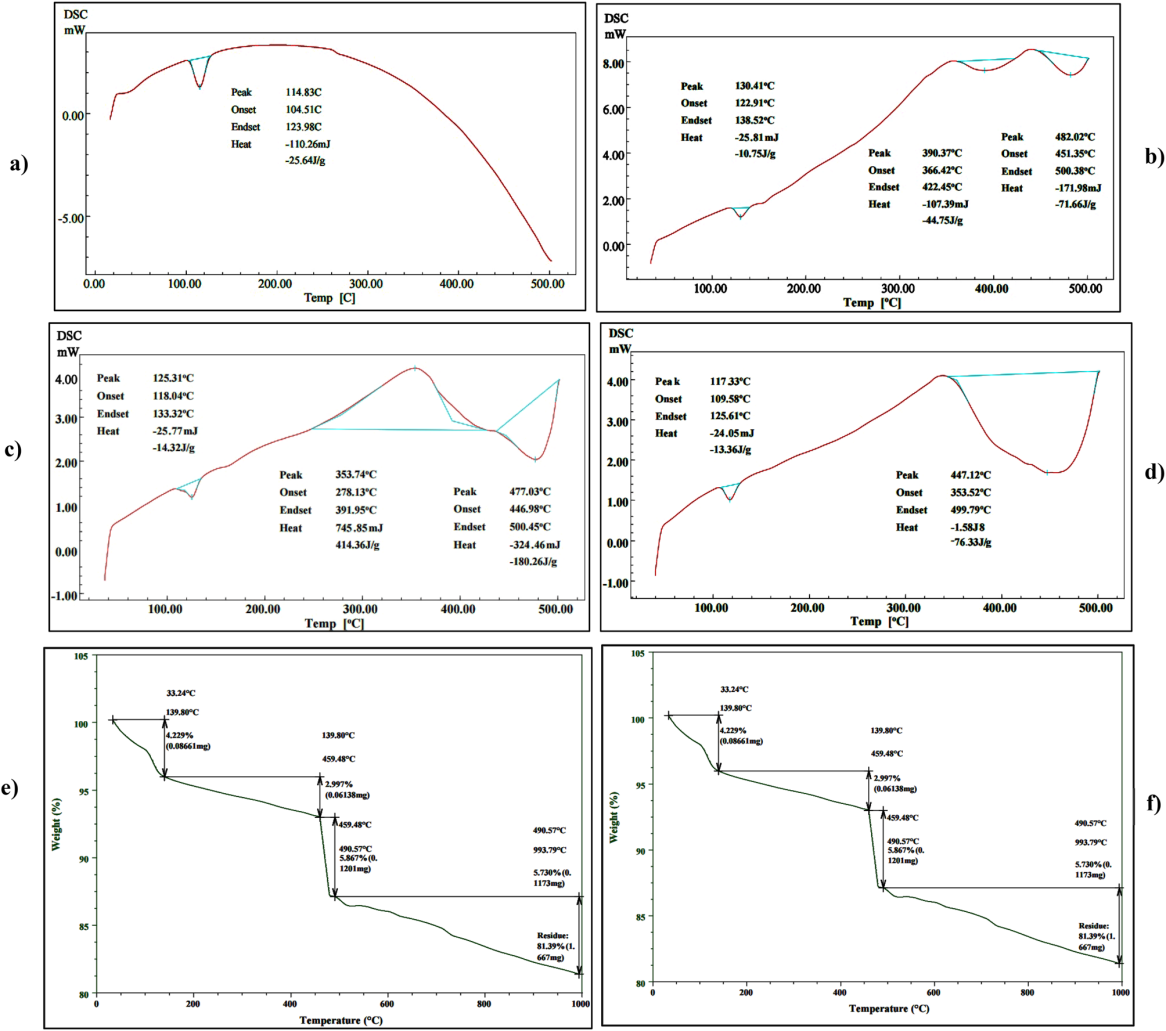
DSC analysis pattern for rock phosphate leftover samples, (**a**) at zero incubation time, (**b**) after 12 h of incubation time, (**c**) after 24 h of incubation time, (**d**) after 32 h of incubation time, (**e**) TGA analysis graph of rock phosphate leftover samples at zero incubation time, and (**f**) after 32 h of incubation tim.

Figure 11a,b,c,d depict DSC curves for residual RP samples at various intervals (0, 12, 24, and 32 h) throughout fermentation time, with a heating rate of 10 °C min^–1^ up to 500 °C in a liquid nitrogen atmosphere. Due to the evaporation of moisture water, all DSC curves showed an exothermic peak that began at 30 °C and terminated at 130 °C. It was noticed at zero time (Fig. 11a) incubation sample showed a peak at 114.83 °C with a heat flow of 1.29 mW mg^–1^, the heat capacity of –110.26 mJ, and enthalpy of –25.64 J g^–1^. On the other side, after 12 h of incubation time samples showed an additional two exothermic peaks at 390.37 and 482.02 °C with heat flow of 7.61, and 7.41 mW mg^–1^, the heat capacity of –107.39, and –171.98 mJ, and enthalpy of –44.75, and 71.66 J g^−1^ were noted as illustrated in Fig. 11b. Mainly oxidizing organic materials caused these peaks in original phosphate due to the fermentation process by *B. haynesii* strain ACP1, which is why the peaks were so high. After 24 h of fermentation, the thermal curve showed little change due to the solubilization process. Two additional exothermic peaks were recorded at 353.74 and 477.03 °C with heat flow of 4.11 and 2.03 mW mg^–1^, the heat capacity of 745.85 and − 324.46 mJ, and enthalpy of 414.36, and − 180.26 J g^−1^, respectively as graphed in Fig. 11c. At the end of the fermentation process (Fig. 11d**)**, a characteristic exothermic peak appeared at 447.12 °C with a heat flow of 1.68 mW mg^–1^, and a heat capacity of − 1.58 J, and enthalpy of –876.33 J g^−1^. In this regard, Dabbebi et al.^32^ proposed that the two oxidation peaks belong to two types of organic materials of Tunisian phosphate washing waste that were pyrolyzed at different temperatures. The study of Bezzi et al.^33^ ascribed that the two endothermic peaks at 71.65 °C and 360.20 °C in the DSC sweeps for the floatation treated samples were attributed to reactions of moisture water evaporation and the composition of organic matter, respectively. The absence of a carbonate peak in the sample treated by floatation indicates that flotation treatment is highly successful for separating the carbonates-phosphates system. As the obtained findings in the current investigation, Aissa et al.^34^ recorded a similar observation pattern of thermal analysis (DSC-TG) data of RP from the Hazm Al-Jalamid region (North of Saudi Arabia).

Thermogravimetric analysis (TGA) was used to evaluate the thermal stability of the leftover RP samples from room temperature to 1000 °C. When the endothermic low-amplitude peak emerges at around 139.80 °C, a weight loss of up to 4.22 percent is seen, corresponding to the dehydration of ions HPO_4_^–2^ according to the following reaction^35^ as illustrated in Fig. 11e.

$$2{\text{HPO}}_{4}^{ - 2} \to {\text{P}}_{2} {\text{O}}_{7}^{ - 4} + {\text{H}}_{2} {\text{O}}$$

It was determined by the TGA (Fig. 11e) that the breakdown of organic materials occurred in the 139.80 °C ≤ T ≤ 490.57 °C intervals. Therefore, the disintegration of mineral carbonates in the RP is responsible for the last 5.73% weight loss between 490.57 and 993.79 °C. On the other side, Fig. 11f illustrates the TGA pattern for the RP residual after 32 h incubation time from the fermentation process. It was noted that the mass loss increased sharply (7.38%) at 35.02 °C and lasted up to 546.51 °C. In the second weight loss of 3.143%, an area of intense endothermic owing to the decomposition of carbonates (calcite (CaCO_3_) and dolomite (CaMg(CO_3_)_2_) with the release of CO_2_ was observed as documented by El Ouardi et al.^36^. As a result of the increase in the thermal decomposition kinetics of carbonates, the last weight loss of 2.212 percent was recorded between 750 and 993 °C, as seen in Fig. 11f. There is an obvious difference in thermal curves of the leftover RP samples throughout the fermentation process compared to that sample taken before the fermentation process, because ACP and acid production by *B. haynesii* strain ACP1 induced hydrolysis and solubilization of organic phosphate portion and hence, led to weight loss in different patterns. These findings were in line with those obtained by other investigators in their study of RP thermal analysis^2,29,37^.

### Fourier-transform infrared spectroscopy (FT-IR) and XRD analysis

**Figure 12:**
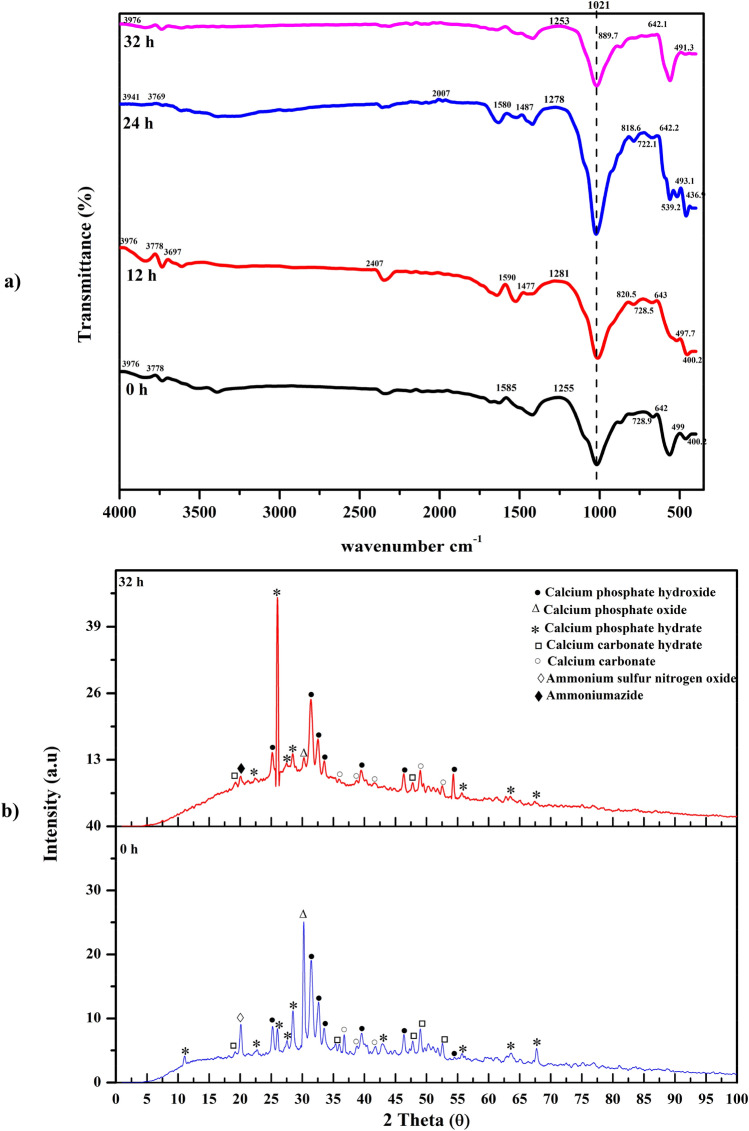
**(a)** FTIR pattern rock phosphate residual samples at zero, 12, 24, and 32 h incubation time and (**b)** XRD analysis chart of rock phosphate leftover samples at zero and 32 h of incubation time.

Apatite's phosphates, carbonates, and OH- hydroxyl ions of leftover RP samples, which were taken before and after the fermentation process, were identified using infrared spectrophotometry as shown in Fig. 12a. FTIR analysis showed that the fermentation and solubilization processes had a noteworthy and substantial influence on the strength and positions of the vibrational bands, as well as the existence and disappearance of some peaks. Furthermore, the band sites and intensities were considerably altered following the solubilization process. In the fingerprint area between 1600 and 400 cm^−1^, several distinct peaks can be detected in various natural or manufactured apatitic phosphates. The leftover RP samples collected after 12, 24, 32 h of incubation time showed absorption peaks at 453, 516–563, 1012–1022 cm^−1^ were assigned to the asymmetric deformation vibration of P=O in PO_4_^3^ as a result of ACP and organic acids action. Moreover, the number of phosphate bands changed as the bend intensified from 667 to 788 cm^−1^. The shit might be caused by the crystal lattice's varying repulsion potential as it contracts or expands. Bachouâ et al.^30^ was documented that asymmetric and antisymmetric elongations of (PO_4_)^−3^ groups account for the bands at 966, 1045, and 1097 cm^−1^, respectively, and other bands (474, 578, and 605 cm^−1^) can be attributed to symmetrical and antisymmetric deformations of (PO_4_)^−3^ groups. Anti-symmetrical vibration of the (CO_3_)^−2^ group is responsible for peaks seen at 870 and 1420–1426 cm^−1^ in calcite and dolomite phases; respectively, this finding agrees with published carbonate data^29^. The intensity of the (CO_3_)^−2^ absorption bands at 728 cm^−1^ showed a consistent trend for 24 h of cultivation time but then disappeared. Carboxylic acid's (COO^−^) vibration is responsible for the absorption peak at 1418 cm^−1^, as well as the peak at 1525 cm^−1^. Their intensity increases as the acid concentration increase^38^. The bands seen in the area of 788 cm^−1^ might be caused by silicate group vibrations. Bands appearing at 672 cm^−1^ are attributable to SO_4_^–2^, and the bending and antisymmetric stretching vibrations of OH^−^ may correlate to the peaks at 1642 cm^−1^. However, water-adsorbed absorption peaks were recorded at 3611 cm^−1^ and 1628 cm^−1^ corresponding to the stretching modes of vibration of H–OH. The absorption peak at 518 cm^−1^ corresponds to the Si–O–Si asymmetric stretching in RP residuals after incubation time ended due to the solubilization process, which aids in leaching Si from the rock matrix. Once the samples were solubilized, the Si–O–Si (*v*_*1*_) and O–C–O (*v*_*3*_) absorption bands became apparent at 539–818 cm^−1^ and 2400 cm^−1^. The removal of the bands associated with calcite at 400 cm^−1^ and carbonate at 1590 cm^−1^ indicated that carbonate and calcite substitutions induce vacancies at the OH sites, and that hypothesized the treatment was responsible for the entire breakdown of carbonate bands and intensity decreases. The obtained results were confirmed by XRD analysis. As part of the study, the crystal phases of the Egyptian natural RP were determined using powder X-ray diffraction (XRD) analysis. X-ray diffraction was used to visualize changes in the mineral structure and arrangement in RPs before and after the fermentation process. The findings verified the occurrence of apatite as a significant mineral.

Phase and crystal structure of the natural RP particles before (at zero incubation time) and after the fermentation process (at the end of cultivation time) were investigated by X-ray diffraction (XRD) study and the results were shown in Fig. 12b. This data was in good conformity with the Joint Committee on Powder Diffraction Standards (JCPDS). The EDS patterns of the RP particles at zero incubation time revealed major peaks at 2θ values of 10.89, 23.59, 26.09, 27.79, 28.86, 43.26, 64.66 and 67.86 with the lattice planes of (001), (− 102), (− 121), (0–21), (1–21), (300) and (4–31), respectively. While at the end of the fermentation process (32 h) values of 2θ were 22.90, 26.50, 28.03, 63.68, and 66.22 indicating the presence of calcium phosphate hydrate. In the RP leftover sample at zero incubation time, a strong major peak was observed at a 2θ value of 30.91 with the plane (100), which confirmed the presence of calcium phosphate oxide. However, this peak was less intense in the RP leftover samples after the fermentation and bio-solubilization process (32 h). Additionally, the RP leftover samples collected at zero incubation time from the cultivation process were characterized by the presence of the major peak at 2θ values of 20.57 with the plane (400). Meanwhile, after 32 h of the cultivation process, it was noticed that the major peak appeared at a 2θ value of 20.49 with the plane (002), which confirmed the existence of ammonium sulfur nitrogen oxide and ammonium azide, respectively. Calcium phosphate hydroxide (hydroxylapatite) was detected in the RP leftover samples that were collected before and after the fermentation and bio-solubilization process with major peaks at 2θ values of 25.86, 31.76, 32.89, 34.05, 39.79, 46.68, and 54.44 with the lattice planes of (002), (211), (300), (202), (130), (222) and (104), respectively but the final peak was very weak in RP before solubilization process at zero incubation time. Furthermore, some other peaks were noticed at 2θ values of 35.97, 39.41, and 41.616 with planes (110), (113), and (131), respectively, which endorsed the occurrence of calcium carbonate (calcite) in the RP before solubilization at zero incubation time. The same results were obtained by Aissa et al.^33^, who reported that calcium carbonate (calcite) in the RP observed major peaks at 2θ values of 35.99 and 39.43. While 2θ values of RP after solubilization were 36.17, 38.61, 41.61, 48.67, and 52.45 with the lattice planes of (200), (022), (131), (202), and (113), respectively. The RP pattern at zero incubation time revealed major peaks at 2Ɵ values of 19.75, 35.68, 47.15, and 52.35 indicating the presence of calcium carbonate hydrate (monohydrocalcite).

## Conclusions

This study emphasized robust microbial engineering approaches for the large-scale production of a newly discovered ACP accompanied by organic acids production from a local bacterial strain isolated from tanning and leather factories wastewater and identified as *B. haynesii* strain ACP1 based on molecular and morphological characterization. *B. haynesii* strain ACP1's solubilization of RP is caused by the ACP and organic acids production. However, extensive study into the exact mechanism and correlation between the two is needed for scientific confirmation. The innovative media formulation for the production of both was achieved through sequential statistical optimization strategies. It was found that glucose, RP, (NH_4_)_2_SO_4_, and NaCl are the most noteworthy variables affecting the production and solubilization processes. The efficiency of ACP productivity was further enhanced through bioprocessing scale-up approaches in 7 L bench-top bioreactors. *B. haynesii* strain ACP1 produced ACP in lockstep with its growth pattern, peaking in the late exponential phase. When compared to the initially started medium, 3.46-fold increments resulted in a substantial improvement in ACP production under uncontrolled pH conditions. It was found that the lactic acid followed by glutamic acid, and hydroxybenzoic acid isomer are represented as the most abundant acids secreted by the strain under investigation. The obtained results of thermal, morphological, and functional characterization of RP remaining samples using TGA, DSC, SEM, EDS, FTIR, and XRD studies emphasize the significant effect of organic acids and ACP activity on the solubilization of RP particles.

## Material and methods

### Sample collection and isolate sources

Wastewater samples were collected aseptically from the different tanning and leather manufactories' sites, Alexandria, Egypt. The samples were transported to the Bioprocess Development laboratory, and stored in the refrigerator at 4 °C until further processing.

### Enrichment and isolation of ACP producing bacteria

The ACP-producing bacteria were enriched as the method described by Abdelgalil using Pikovskaya’s broth medium^39^; supplemented with RP, instead of Ca_3_(PO_4_)_2_, pH 7.0 under incubation of 50 °C with 200 rpm for 72 h. The RP was obtained from El-Nasr Phosphate Company, which originated from the Abu-Tartur (Western Desert), and passed through a 0.125-mm sieve. The Luria–Bertani (LB) medium agar was used to isolate, purify, and maintain the enriched bacterial isolates, as illustrated by Abdelgalil^40^.

### Qualitative screening for ACP activity

The phosphate-solubilizing and organic acids production activities were investigated using Pikovskaya’s agar supplemented with CaCO_3_, while ACP activity was assessed using chromogenic substrates like para-nitrophenyl phosphate (*p*NPP) agar media as a screening media^41^. The appearance of the clear zone around the colonies on PVK agar plates and the appearance of district visible yellow staining colonies indicated positive acid and ACP production capabilities. Therefore, a promising isolate showing the highest solubilization clear zone and the highest color intensity was designated as ACP1, and it was picked up for further study and was subjected to further morphological and molecular identification.

### Quantitative screening for ACP activity

The cell-free supernatant that was harvested after cultivation of the one mL of activated pre-culture of the selected isolates on the fifty mL of PVK broth medium dispensed in 250 mL Erlenmeyer flask with Egyptian RP (2%) at 50 °C, 200 rpm for 72 h, was used as crude enzyme for a quantitative assessment of ACP activity. ACP activity was assayed in a standard reaction mixture containing 100 mM sodium acetate buffer pH 4.0, 1 mM *ρ*NPP [Sigma Chemical Co.], and the enzyme solution. The color development was measured spectrophotometrically at 405 nm (ε = 17,800 M^−1^ cm^−1^) using a UV–Visible spectrophotometer after incubation time ended at 65 °C. One unit of ACP activity has been expressed as an international unit (U), it represents the amount of enzyme that produces 1.0 μmol *ρ*-nitrophenol in 1 min at pH 4.0 and 65 °C; the activities were expressed in U L^−1^ min^−1^.

### Amplification of the 16S rRNA gene, sequencing, and similarity

The salting-out method was followed to extract the total genomic DNA of the most potent ACP-producing isolate. Afterward, the amplification of the fragments of the *16S rRNA* gene by the PCR reaction, using universal forward primer 5′-AGAGTTTGATCMTGGCTCAG-3′ (corresponded to position 8 of *Escherichia coli* *16S rRNA*) and reverse primer 3′-TACGGYACCTTGTTACGACTT-5′ (corresponded to position 1514 of *Escherichia coli* *16S rRNA*). The PCR reaction mixture consisted of (1.5 μl) 10 pM for each primer, 0.1 μg of chromosomal DNA, (5 μl) 2 mM dNTPs, (0.4 μl) 2U of Taq polymerase, (5 μl) 1 × polymerase buffer (Fermentas, Germany) and 36.8 μl of nuclease-free water for a 50 μl reaction. PCR amplification was performed using a thermal cycler (Multigene Optimax, Labnet International, Inc.) under the following conditions: 4.0 min at 95 °C, then 30 cycles at 94 °C for 1.0 min, 55 °C for 1.0 min, and 72 °C for 2.0 min, followed by an additional 10 min at 72 °C as a final extension step. Sequencing, and the construction of a neighbor-joining phylogenetic dendrogram, were executed according to the method of Abdelgalil et al.^42^.

### Morphological investigation of *B. haynesii* strain ACP1

The morphology of the cell surface and the shape of the most potent ACP-producing bacteria were shown using a scanning electron microscopy (SEM) unit at the City of Scientific Research and Technological Application's (SRTA-city) laboratory center. Using a sputtering device (JFC-1100 E Joel, USA), the dried bacterial thin film was covered with a thin coating of gold.

### Optimization of the physical parameters for the production of ACP

Three key fermentation physical parameters were studied individually by the quasi-optimum protocol (One variable at a time approach-OVAT) to investigate the optimum fermentation parameters of bacterial culture for the best ACP activity. For various physical variables, such as temperature, pH values, and initial inoculum size, the effectiveness of ACP output was assessed by methods as described by Abdelgalil^40^. A standard inoculum 2% aliquot of activated pre-culture of the most efficient strain was used for inoculation of PVK broth medium supplemented with 2% RP^39^ followed by incubation at different temperatures (45 °C, 50 °C, and 55 °C) for 48 h using a rotary shaker (200 rpm) to investigate the influence of different temperature on the ACP production process. While, the different activated inoculum sizes of the selected strain (1, 2, 3, 4, 5, 10%) were exploited as pre-inoculum for aerobic cultivation of PVK broth medium supplemented with 2% RP at 50 °C, 200 rpm to select the most effective inoculum size in ACP production process. The most effective temperature, and inoculum size of the most promising strain were exploited to investigate the most optimized pH for ACP production. The initial pH value of 50 mL PVK broth medium supplemented with 2% RP in 250 mL Erlenmeyer flasks was adjusted individually to 3.0, 4.0, 5.0, 6.0, 7.0, and 8.0 using 0.1 M HCl. The initial pH value of 2% RP-PVK broth medium without any pH adjustment was also assessed. The inoculated flasks were incubated aerobically at 50 °C for 48 h in a rotary shaker incubator (200 rpm). The cell-free supernatants of all experiments were used to determine ACP activity.

### Media formulation for ACP production by *B. haynesii* strain ACP1

#### Plackett and Burman design (PBD)

The significant independent variables affecting the production peak of the ACP were assessed using 2^k^-PBD (variables, k = 11) with four central points in 16 combination trial batches (twelve main batches plus four central point batches). The eleven independent variables were picked up randomly for PBD, namely glucose, potassium citrate, sodium nitrate (NaNO_3_), ammonium sulfate (NH_4_)_2_SO_4_, urea, RP, sodium chloride (NaCl), magnesium chloride (MgCl_2_•6H_2_O), cobalt chloride (CoCl_2_•6H_2_O), copper sulfate (CuSO_4_•5H_2_O), and nickel sulfate (NiSO_4_•H_2_O). They were investigated to identify the most significant factors for the peak production of ACP by identifying *B. haynesii* strain ACP1 in submerged fermentation, and ACP activity was chosen as a response with a confidence level of all intervals at 95% to generate regression coefficient values. Each independent variable was investigated on two levels: high (+ 1) and low (− 1) to assess its influence. The four central points were also utilized in four distinct batches for all of the independent variables Table 1. The obtained experimental data were subjected to statistical analysis involving ANOVA, determination of coefficients, and the polynomial model reduction via the essential experimental design free software programs to evaluate the efficiency and effectiveness of the regression model. For mathematical screening modeling, the following first-order polynomial approach was employed:

$$Y = \beta_{0} + \mathop \sum \limits_{i = 1}^{k} \beta_{i} X_{i}$$

where *Y* is the predicted response (ACP activity U L^−1^ min^−1^), *β*_*o*_ is the model intercepts, *β*_*i*_ is the linear regression coefficient, and *X*_*i*_ is the coded independent variables estimates. The findings were analyzed based on each variable's influence. The influence on ACP throughput of each specified variable was determined using the following equation:

$$E\left( {x_{i} } \right) = \frac{{2\left( {\sum Y_{i}^{ + } - Y_{i}^{ - } } \right)}}{{\text{N}}}$$

where *E* (*x*_*i*_) = the response value effect of the investigated variable (*x*_*i*_), *Y*_*i*_^+^ and *Y*_*i*_^−^ = ACP throughput efficiency achieved for each variable at high and low values, respectively; and N = the number of trials. The sign of the effect reflects the extent of further improvement it is considered. The greater confidence variables had been taken into account as influencing the response or the output variable. In terms of ACP productivity, factors with the greatest *t*-value and confidence levels above 95% (*p* < 0.05) were regarded as highly impactful. The categorization of the independent variables by their significant productivity implications has been achieved by the Pareto diagram construction. The obtained results from the Pareto diagram revealed that the independent variables like *X*_*1*_, glucose; *X*_*4*_, (NH_4_)_2_SO_4_; *X*_*6*_, RP; and *X*_*7*_, NaCl exhibited the highest positive effects on ACP throughput. A verification experiment was undertaken to evaluate and compute the average ACP production for the predictable optimum levels of the independent variables.

### Response surface methodology (Rotatable Central Composite Design)

The four most influential independent variables were chosen for further optimization as a consequence obtained after conducting PBD for screening of independent variables and identifying the optimum value of every independent variable that would give the highest ACP output. ACP activity (*Y*) has been chosen from PBD to be optimized as a dependent response variable and the objective was to get the highest possible ACP activity. The correlation of four significant independent variables (glucose, *X*_*1*_; (NH_4_)_2_SO_4_, *X*_*2*_; RP, *X*_*3*_; and NaCl, *X*_*4*_) and the dependent variable (ACP activity, *Y*) was investigated through RCCD at five different levels denominated as (− α, − 1,0, + 1, + α), where α = 2. The total number of treatment combinations according to this approach is $2^{k} + 2k + n_{0}$; where *n*_*0*_ is the number of repetitions of the experiments at the center point and ‘*k*' is the number of independent variables. The variables *X*_*i*_ have been coded as *x*_*i*_ in accordance with the following transformations for statistical calculations:

$$x_{i} = \frac{{X_{i} - X_{0} }}{\delta X}$$

where *x*_*i*_ is the dimensionless coded value of the variable; *X*_*0*_ is the value of the *X*_*i*_ at the center point and *δX* is the step change of the real value of variable *i* representing a variation of a unit for the dimensionless value of variable *i*. A 2^k^-factorial central composite design was performed to build experiments with sixteen cube points plus six center points and eight axial points as shown in Table 3 for further optimization of the four medium variables that exhibited significant positive effects on ACP production by *B. haynesii* strain ACP1 and had the highest percentage of contribution. In ANOVA, Fisher's statistical analysis and *p*-value were used to investigate the influence of each independent variable on responses and model competency. A second-order polynomial equation was used to analyze the peak's area, and the data were fitted using a multiple regression model. The following quadratic polynomial equation depicts the statistical relationship between the ACP activity (*Y*) and the selected independent variables:

$$Y = \beta_{0} + \mathop \sum \limits_{i} \beta_{i} X_{i} + \mathop \sum \limits_{ii} \beta_{ii} X_{i}^{2} + \mathop \sum \limits_{ij} \beta_{ij} X_{i} X_{j}$$

where *Y* is the predicted response (ACP activity U L^−1^ min^−1^); *β*_*0*_ is the model intercept; *X*_*i*_ and *X*_*j*_ are the independent variables, *β*_*i*_ is linear coefficients; *β*_*ij*_ is the cross-product coefficients; *β*_*ii*_ is the quadratic coefficients. Multiple determination coefficients (*R*^*2*^) and the lack-of-fit value can explain the model's appropriateness. The statistical model was validated concerning ACP production under the conditions predicted by the model in shake flask conditions to evaluate the equation model and ensure that the theoretical values of each variable are accurately computed.

For optimizing and determining interaction coefficients across many parameters the analysis of contour and surface plots achieved was utilized. The 3D response surface plots were built via the STATISTICA software package to determine interactions among the significant variables and their impact on the response (*Y*, ACP activity). The approach of points prediction was utilized to establish optimal values for each variable. Contour plots and surface response plots are diagrammatic representations of response values. These diagrams help project the magnitude of each variable's and interactions' influence. The essential experimental design-free software was exploited in the present study to analyze the obtained data via multiple linear regressions.

### Bioprocess strategies for scaling-up production of bacterial ACP

The evaluation of *B. haynesii* strain ACP1 growth kinetics in a submerged cultivation system was targeted in the present study by strategically scale-up the ACP production process from shake flask scale to bench-top bioreactor scale.

### Shake-flask batch cultivation

In a shake flask, the standard batch fermentation mode approach was followed. A revitalized culture plate of *B. haynesii* strain ACP1 was used to prepare 10 h pre-cultured inoculum in LB medium, a 5% (v/v) activated pre-cultured inoculum was added to inoculate a 250 mL Erlenmeyer flask contained 50 mL of the optimized medium [(w/v%); glucose, 2.32; (NH_4_)_2_SO_4_, 0.13117; RP, 4.16; NaCl, 0.1343; without pH adjustment]. The inoculated flasks were incubated in an orbital shaker at ± 50 °C and 200 rpm for 32 h, and the bacterial culture samples were drawn out periodically after two h throughout the incubation time. Actively growing bacterial cells were tracked by spectrophotometric monitoring their absorption at 600 nm against optimized medium solution as a blank. The cell-free supernatant was obtained by the centrifugation of bacterial culture aliquots at 10,000 rpm for 15 min under cooling conditions. After centrifugation, the different analytical parameters like ACP activity, glucose concentration, total soluble phosphate concentration, and total soluble protein concentration throughout the incubation period were monitored. All experiments were executed in triplicate.

### Stirred-bioreactor batch cultivation system

Production ACP was scaled up in a 7-L bench-top bioreactor with a 4.0 L working volume (Bioflow 310, New Brunswick, N J, USA) as documented by Abdelgalil^40^. The bioreactor was equipped with monitors to measure and control the variables such as foam, temperature, pH, agitation rate, and dissolved oxygen. The bioreactor vessel was equipped with two 6-bladed disc-turbine impellers and four baffles. The bioreactor was outfitted with an air compressor unit that initially supplied compressed air at 0.125 VVM (air volume per broth volume per minute) through a sterile filter. A bio-command multi-process control software supplemented by a control panel of 10.4 color touch-screen interface computer system was utilized to automate the process. pH uncontrolled batch cultivation mode was started by aseptically inoculating the sterile statistically optimized medium (unadjusted pH 7.55) content bioreactor vessel by 4.0% of the log phase revitalized pre-cultured inoculum. The pH of the fermentative system fell under an uncontrolled process, while temperature, and agitation rate were maintained at 50 °C and 200 rpm, respectively. An antifoam agent (silicone oil 0.5:10 v/v) at a concentration of 1:100 (v/v) in distilled water was applied to inhibit foam development during the fermentation process. While the pH-controlled batch cultivation mode was initiated with conditions as recorded in pH uncontrolled batch cultivation mode unless the pH of the process was controlled by automatic feeding of 2 N NaOH and 2 N HCl to pH 7.5.

Through the fermentation process (32 h), 20 ml of culture samples were withdrawn regularly at various time intervals in pre-weighed, 50 ml sterile falcon tubes. A track of the cell growth using a Beckman DU spectrophotometer was computed to demonstrate light absorbance at 600 nm against blank. A centrifugal process at 10,000 rpm for 15 min was used to collect the cell-free supernatant and utilized for further analytical analysis, whilst the collected cell pellets were used for dry cell biomass assessment. An integrated model of bioreactor hydrodynamic and microbial kinetics is necessary to analyze and optimize the performance of a microbial process.

### Analytical procedures

#### Determination of dry biomass weight.

Gravimetric determination of the dry biomass was performed by centrifugation of withdrawn samples collected in pre-weighed sterile falcon tubes throughout the fermentation process at 10,000 rpm for 10 min. The distilled water-washed cell pellets were subjected to heat in an oven at 80 °C overnight, cooled down in a desiccator, and weighed. The linear correlation between the dry biomass weight and the bacterial culture's optical densities (OD _600_) was built to determine the correlation factor (*δ*).

#### Determination of glucose Concentration.

The glucose concentration in culture filtrate samples was analyzed spectrophotometrically using an enzymatic colorimetric kit (Diamond Diagnostics, Egypt).

#### Determination of total soluble phosphate content.

The spectrophotometric measurement of accessible phosphorus in the culture filtrate was done using the ascorbate method^43^. To determine the concentration precisely, the intensity of the molybdenum-blue color was measured at 820 nm, and the standard curve was plotted using 100 g mL^−1^ of potassium dihydrogen phosphate solution stock.

### Protein concentration assay

Total extracellular protein concentration was assessed by Lowry’s methodology for cell-free supernatant collected samples (in triplicate). A stock solution (1 mg mL^−1^) of bovine serum albumin (BSA, Sigma) was used to construct the standard curve^44^.

### Morphological structure and energy-dispersive spectroscopy (EDS) analysis of the RP

A scanning electron microscope (Jeol jsm 6360 LA, Japan) coupled to an in-situ EDS spectrophotometer at the SRTA-city laboratory center was used to study the surface morphology of RP leftover samples as well as their energy-dispersive X-ray patterns before and after submerged cultivation of bacterial cells.

### Detection of organic acid production

Liquid chromatography-tandem mass spectrometry (LC–MS/MS) was used to identify and quantify the organic acids produced by the strain under investigation. The organic acids were quantified using a Luna® 3 µm HILIC column (100 × 4.6 mm) from Phenomenex in the United States. A 0.1% of HCOOH acid in water (mobile phase A) and 0.1% HCOOH acid in methanol (mobile phase B) were used as mobile phases. The column was maintained at 40 °C and used in gradient elution mode with a flow rate of 0.35 mL min^−1^ and a 20 µL injection volume. The individual primary stock solutions of the selected organic acids (1.0 mg mL^−1^) were prepared in a water–methanol mixture (80:20, *v v*^−1^) and stored at 4.0 °C for one month. Appropriate dilutions were made with a water–methanol mixture (80:20, *v v*^−1^) to produce a combined intermediate stock solution of pyruvic acid (50 µg mL^−1^), lactic acid (10 µg mL^−1^), gluconic acid (1.5 µg mL^−1^), glutamic acid (1.2 µg mL^−1^) tartaric acid (1 µg mL^−1^), citric acid (0.5 µg mL^−1^), maleic acid (0.4 µg mL^−1^), salicylic acid (0.4 µg mL^−1^), and succinic acid (0.3 µg mL^−1^). The intermediate stock solution was further serially diluted to produce standard working solutions that cover the linearity ranges tabulated in Table S1. 20-µL of these working standard solutions were determined by the proposed LC–MS/MS method. In the same manner, the quality control samples at three concentration levels were prepared in the water–methanol mixture (80:20, *v v*^−1^): low-quality control (LQC), medium-quality control (MQC), and high-quality control (HQC), as illustrated in Table S2.

### Solid-phase extraction (SPE) and clean-up of the samples

A non-sterile single-use cellulose syringe filter (0.45 µm) was used to remove particulates from the samples. One milliliter of each sample filtrate was diluted with 4 mL of 25 mM ammonium acetate, and the pH was adjusted at (6–7) by 1% ammonium hydroxide. The recommended procedures offered by the manufacturer of the SPE cartridge were implemented. After conditioning and equilibrating the SPE cartridge (Strata X-AW, 200 mg 3 mL^−1^), the diluted sample was loaded, followed by washing with 1.0 mL 25 mM ammonium acetate (pH 6–7) and 1 mL methanol. Then, the acids were eluted from the SPE cartridge using 1 mL of 5% ammonium hydroxide in methanol. The eluted solution was evaporated to dryness at 40 °C under a stream of nitrogen and reconstituted in 5 mL of a water–methanol mixture (80:20, *v v*^−1^), and 20-µL of this solution was injected using the proposed LC–MS/MS method.

### Instrumentation and software

The UHPLC analysis was carried out using an Eksigent ekspert™ ultraLC 100 system (Dublin, California, USA), which comprises two linked pump units, one with an integrated degasser and the other with a mixer, refrigerated auto-sampler, and column oven chamber. SCIEX QTRAP® 5500 (SCIEX instruments, Foster City, Canada) was used in multiple reaction monitoring (MRM) to obtain mass spectrometric detection. Under negative ionization modes, a Turbo V™ electrospray ionization (ESI) interface was employed. The UHPLC and the mass spectrometer were controlled using Analyst® software version 1.6.2. MultiQuant 3.0 was used to perform post-run data processing. The concentration of samples was achieved using a TurboVap® LV evaporator under nitrogen from Biotage GB Limited, UK.

### Chromatographic conditions and quantitative MRM procedure

For the quantitative monitoring of the targeted organic acids, a negative MRM scanning mode was utilized. In addition, 20 to 50 ng mL^−1^ of the targeted organic acids in methanol were directly infused in the mass spectrometer using a gas-tight syringe at a flow rate of 7 µL min^−1^ to optimize mass spectral data. Tables S3 and S4 show the optimized MRM transitions of the selected organic acid and the critical operational parameters of the ion source of the mass spectrometer, respectively. The ESI–MS/MS spectra of targeted organic acids with proposed fragmentation patterns are shown in Figures S1–S10. For the quantitative analysis, Table S5 shows the complete chromatographic elution procedures.

### Method validation

The LC–MS/MS procedure validation was carried out according to the current Food and Drug Administration (FDA) guideline on bioanalytical method validation^45^ and the International Conference on Harmonization (ICH) guideline on validation of analytical procedure^46^. Further details on the validation procedures are illustrated in the supplementary file.

### Atomic absorptions analysis (AAS)

The cell-free supernatants and RP leftover samples before and after the cultivation process were exposed to AAS (Zeenit 700 Analytik Jena, Germany) at the laboratory center at the SRTA-City to assess residual amounts of heavy metals.

### Differential scanning calorimetry (DSC) analysis

The residuals of RP samples before and after the cultivation process were subsequently installed in an aluminum sample panel and then, subjected to a differential scan calorimeter (60–A^o^) at the SRTA-city laboratory center to estimate its pyrolysis pattern. The study was done with a heating rate of 10 °C min^−1^ and, a flow rate of 30 ml min^−1^ in a liquid nitrogen environment. The thermogram was obtained from 25 to 500 °C.

### Thermogravimetric analysis (TGA)

The thermal properties of RP leftover samples were studied by the thermogravimetric analyzer (TGA, Model 50/50H, Shimadzu, Japan) at the SRTA-city laboratory center. TGA analysis was achieved under a nitrogen atmosphere (flow rate 20 mL min^−1^), while the temperature was raised gradually from 10 to, 1000 °C with a constant heating rate (10 °C min^−1^). The chart was plotted as temperature versus weight loss (percentage).

### Fourier-transform infrared spectroscopy (FT-IR)

Shimadzu FTIR-8400 S, Japan at the SRTA-city laboratory center, has determined the active chemical bonds or functional groups associated with samples of RP residuals before and after the fermentation process. The KBr disk method was used as a matrix; the spectrum was scanned at a resolution of 4.0 cm^−1^ in the mid–IR range between 4700 and 400 cm^−1^.

### X-ray diffraction (XRD) analysis

The identification of crystal structure, phase, and texture of RP leftover samples was evaluated using an X-ray diffractometer (Bruker MeaSrv [D2-208219]) with CuKα (k = 1.54A°) radiation and scanned between 2θ angular range of 2° to 100° with a scan speed of 0.02°/s.

## Supplementary Information

Supplementary Information.

## Data Availability

All data produced during this study are included in this published article.
